# Molecular interactions of the Na_V_1.5 C-terminal domain: CaM sequestered the IQ motif from the CTD

**DOI:** 10.1016/j.jbc.2025.110871

**Published:** 2025-10-30

**Authors:** Rita F. Gyawu, Torien M. Beard, Svetlana Tikunova, Ali Ulker, Sara A. Garcia, Garrett M. Knotts, Taylor A. Agee, Md Nure Alam Afsar, Brett Kroncke, Jarrod Smith, Jonathan Davis, Christopher N. Johnson

**Affiliations:** 1 Department of Chemistry, Mississippi State University, Starkville, Mississippi, USA; 2 Department of Physiology and Cell biology, College of Medicine, The Ohio State University, Columbus, Ohio, USA; 3 Vanderbilt Center for Arrhythmia Research and Therapeutics, Vanderbilt University Medical Center, Nashville, Tennessee, USA; 4 Center for Structural Biology, Vanderbilt University, Nashville, Tennessee, USA

**Keywords:** voltage-gated sodium channel C-terminal domain (NaV-CTD), calmodulin, calcium, NMR, IQ motif, domain swap configuration, stopped-flow kinetics

## Abstract

Voltage-gated sodium channels (Na_V_) are important for life. Alterations to the synchronized timing of ion conduction can create life-threatening conditions. How Na_V_ conduction responds to changes in intracellular Ca^2+^ concentration has been the subject of extensive investigation. Crystal structures of the cardiac Na_V_ (Na_V_1.5) cytosolic components were reported as trimeric complexes that are restructured in the presence of Ca^2+^. These results formed the basis for a gating model where two Na_V_1.5 molecules interact to alter function in response to Ca^2+^ concentration. Here, we investigated the binding site surface of these trimeric interactions in solution. Nuclear magnetic resonance spectroscopy demonstrated that these trimeric complexes do not form in solution. Analysis of the available structural data indicated that the Na_V_1.5 IQ motif can only accommodate interaction with one protein at a time. Our nuclear magnetic resonance spectroscopy data were consistent with the Na_V_1.5 C-terminal domain (CTD) and the Ca^2+^- sensing protein calmodulin (CaM) engaging the same binding site surface of the Na_V_1.5 IQ motif. Titrations of IQ motif peptide into a 1:1 mixture of ^15^N CTD: CaM sample revealed the Na_V_1.5 channel is biophysically distinct from neuronal Na_V_1.2 as Ca^2+^ enhanced CaM's ability to sequester the Na_V_1.5 IQ motif from the Na_V_1.5 CTD. Stopped-flow kinetic measurements quantified Ca^2+^ release rates from the CaM-IQ and CaM-inactivation gate (IGATE) complexes to provide insight into complex lifetimes. Our work advanced understanding the molecular machinery that underlies Na_V_1.5 gating (CTD-IGATE and CTD-IQ motif interactions) and provided insight into the structural details of CaM-facilitated Na_V_1.5 modification (CaM-IQ motif and CaM-IGATE interactions).

Voltage-gated sodium channels (Na_V_) open in response to membrane depolarization; conduction of Na^+^ ions through the cell membrane generates the initial upstroke of an action potential. The timing of transitions between conducting and non-conducting configurations is essential for proper function (1). Alterations to function of the cardiac Na_V_ (Na_V_1.5) can have dangerous consequences to health (2, 3, 4). The bulk of the channel is contained within the alpha subunit, which is comprised of 24 transmembrane helices arranged into four domains. Similar to a transmembrane domain, a fifth set of helices form a globular structure within the cytosolic C-terminus of the channel. Several of the cytosolic components within the Na_V_ alpha subunit (C-Terminal Domain (CTD), IQ motif, and inactivation gate (IGATE) (defined below)) are dynamic, and these can allosterically modify amino acids that control ion gating (IFM latch, latch receptor, selectivity filters; or activation gate). Reminiscent of the L-type calcium channel (5), the Na_V_ family contains amino acids that were posited to form an EF-hand like motif (helix-loop-helix) in the cytosolic CTD (6). CryoEM and solution nuclear magnetic resonance spectroscopy (NMR) spectroscopy demonstrated that the CTD can interact with the channel IGATE (7, 8). Fluorescence, NMR, and MD simulations also supported an interaction between the IQ motif (discussed below) and the CTD (9, 10, 11). The CTD appeared to use the same binding site (helix I-IV) for interaction with the IGATE and IQ motif (8, 10, 11, 12).

Our recent modeling efforts focused on improving molecular descriptions of the intact Na_V_1.5 alpha subunit in a lipid bi-layer with explicit salt and water (11, 12) (Fig. 1). These simulations leveraged the intact models generated by AlphaFold (https://alphafold.ebi.ac.uk) and improved the predictions of the backbone torsion angles for the majority of the cytosolic amino acids. Notably, our simulations captured dissociation of the CTD from the channel IGATE. Electrostatic contacts unzipped and reformed to create a "walking motion". This translocated the CTD as it shifted out and down to expose the helix-I-helix-IV binding interface for additional interaction (12). Our subsequent MD simulations predicted that the unbound CTD could form a stable complex with the IQ motif (within the context of an intact channel in a lipid bilayer) (11). Interestingly, the CTD-IGATE interaction that dissociated was primarily comprised of electrostatic contacts (8, 12), while the CTD-IQ motif that held together during the 4 × 1 μ sec simulations was predominantly driven by hydrophobic interactions (10, 11).

**Figure 1:**
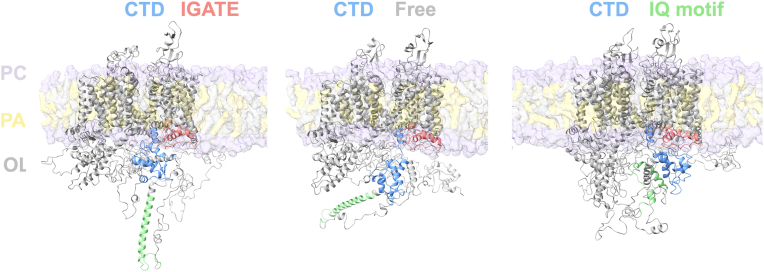
**All atom models of Na_V_1.5 alpha subunit provided insight into the C-terminal domain (CTD) intramolecular interactions (Knotts *et al.* 2024, Lile *et al.* 2025).** Three distinct configurations have been identified with energetic minima in these MD stimulations. CTD bound to inactivation gate (IGATE) (*left*), CTD dissociated from IGATE (*middle*), and CTD bound to IQ motif (*right*) are shown as ribbon models with components color coded: CTD in *blue*, IQ motif in *green* and IGATE in *pink*. Each model contains 2016 amino acids in a 1-palmitoyl-2-oleoyl-sn-*glycero*-3-phosphocholine lipid bilayer with explicit salt and water. Models were generated using 4 × 1 micro-second Amber 23 MD simulations using ff19sb and lipid21 forcefields with the optimal point charge water. Lipid bilayer was comprised of Palmitoyl and Oleoyl and Phosphatidylcholine components. CTD, C-terminal domain; IGATE, inactivation gate.

In 2002, Tan *et al.* demonstrated that cytosolic calcium (Ca^2+^) can modify the function of Na_V_1.5 (13). Given similarities among the Na_V_ family and their prominent role in excitable cells and disease (14) (loss of function variants have been associated with Brugada Syndrome (15) and gain of function variants associated with Long QT syndrome (4)), significant efforts have been put forth to identify and describe protein interactions that can modify channel function. Many structure-function investigations have focused on components of Na_V_ and their interactions with the Ca^2+^-sensing protein calmodulin (CaM) (10, 13, 16, 17, 18, 19, 20, 21, 22, 23, 24, 25, 26, 27, 28, 29) (Fig. 2 and Table S1). An IQ motif (IQxxx[R,K]Gxxx[R,K]) sequence (30) located within the cytosolic helix VI of the channel C-terminus can interact with CaM in both the absence and presence of Ca^2+^ (Fig. 1 and Table S1) (9, 10, 19, 20, 21, 24, 25, 26, 27, 31, 32). Several investigations have also characterized and described aspects of CaM interacting with an IQ motif that was connected to the CTD. Crystallographic studies have provided structures of these CaM complexes in both the absence and presence of Ca^2+^ and fibroblast growth factors (25, 26, 27, 31, 32). Observed contacts between the Na_V_1.5 IQ motif and a neighboring Na_V_1.5 CTD molecule form the basis of a model that explained Ca^2+^ modification of Na_V_ function (27). In this model, the presence of Ca^2+^ restructured CaM's interaction on the IQ motif and the CaM-C domain shifts from the IQ motif to the IGATE (27, 33).

**Figure 2:**
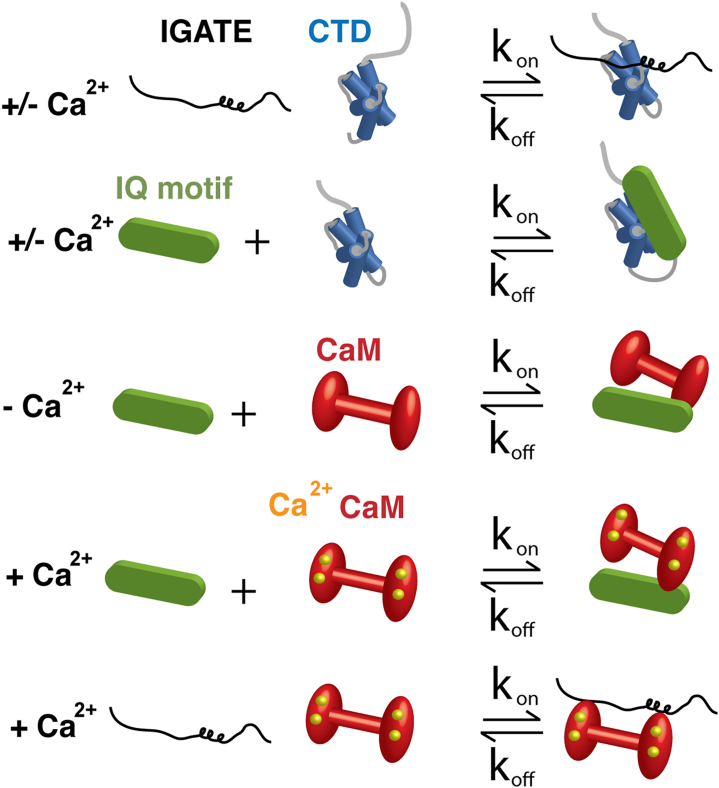
**Cartoon diagram of Na_V_ components that highlight cytosolic Na_V_-Na_V_ and Na_V_-CaM interactions in the absence or presence of Ca^2+^.** Cartoon of: IGATE (*black*), CTD (*blue bundle of four helixes*), IQ motif (*green*), CaM (*red dumbbell*) and Ca^2+^ (*yellow spheres*). Biophysical details of these interactions are provided in Table S1. CaM, calmodulin; CTD, C-terminal domain; IGATE, inactivation gate.

Biophysical characterization of recombinantly expressed components of Na_V_1.2 demonstrated that Ca^2+^ restructured the CaM-IQ motif interaction (switched from parallel to anti-parallel orientation) and a reduced affinity was observed in the presence of Ca^2+^ (34, 35). This reduction in CaM affinity supported a Na_V_1.5 "handoff" model where the IQ motif was posited to function as a molecular switch. In this model, a CTD-IQ motif interaction released CaM for interaction with other parts of Na_V_1.5 (36), such as the IGATE (18). Isothermal titration calorimetry (ITC) of recombinantly expressed components (CTD-IQ motif, CaM, CTD, IQ motif, CaM-N and CaM-C) formed the basis for a bridging mechanism where CaM spanned from the IQ motif to the IGATE (19). Early studies captured portions of the two-site CaM-IGATE binding interface and observed modest μM affinities (18, 19, 29). We subsequently demonstrated that CaM can directly engage a construct corresponding to the full-length IGATE with nM affinity (28). This high affinity CaM-IGATE interaction supported a mechanism of CaM transitioning both the -N and -C domains from the IQ motif to the IGATE (28, 37). Similarly, an extended Na_V_1.5 IQ motif peptide enhanced CaM binding affinity in the presence of Ca^2+^; however, in the corresponding crystal structure (PDB ID 6MUD), the CaM-N domain failed to engage the IQ motif. Conversely, a Na_V_1.4 CaM-N-IQ motif interaction was captured by crystallography. These data led to a mechanism that explained differences in how Na_V_1.4 and Na_V_1.5 responded to Ca^2+^ (25). The molecular details governing the mechanism of CaM transition from the Na_V_1.5 IQ motif to the IGATE are unclear (37). There is still controversy in the biophysical data as to how these interactions assemble and contribute to a Ca^2+^ sensing apparatus that modifies Na_V_ gating (21, 22, 23, 26, 37, 38, 39). Specifically, it is unclear if and how CaM transitions one or both domains from the IQ motif to the IGATE.

Here, we investigated the posited crystallographic trimeric complexes (CTD-IQ motif-CaM) using solution NMR to understand their role in the transition of CaM from the IQ motif to the IGATE upon Ca^2+^ binding. Specifically, we sought to gain insight into the respective (CTD-IQ motif-CaM) complex lifetimes in the absence and presence of Ca^2+^. Our NMR data revealed CTD-IQ motif-CaM trimeric contacts do not form in solution. Instead, these likely arose from crystallographic domain swap interactions. We then considered that these CaM interactions occur while Ca^2+^ concentrations ([Ca^2+^]) are continually oscillating within a cardiomyocyte. Therefore, we quantified the kinetic Ca^2+^ release rates of the CaM-IQ motif and CaM-IGATE complexes to gain insight into their respective complex lifetimes.

## Results

### Solution NMR spectra provide signatures for protein complexes that are consistent with literature

**Figure 3:**
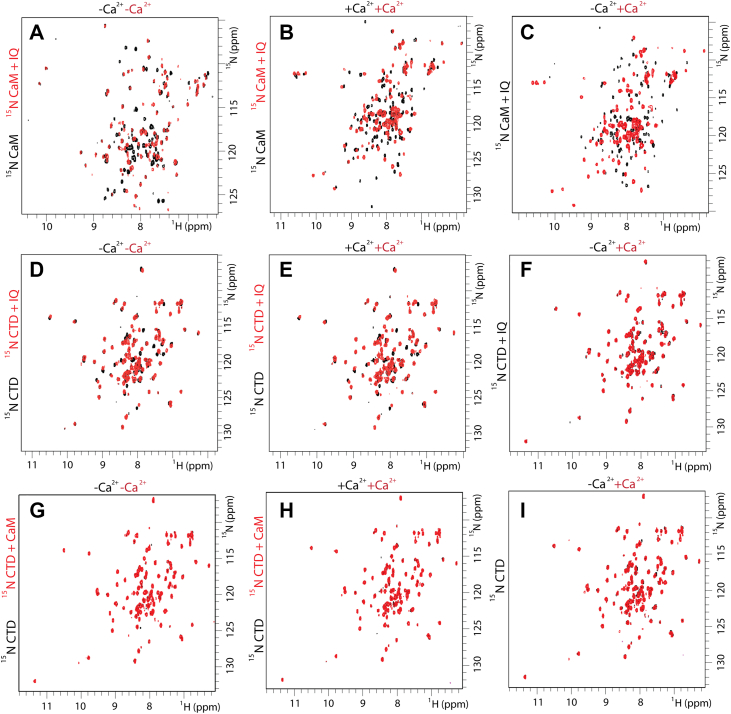
**NMR spectra provide a signature for complex formation.** Overlays of ^1^H-^15^N HSQC spectra of isotopically enriched ^15^N CaM (A–C) or ^15^N CTD (D–I) in the presence of IQ motif peptide, or unlabeled (^14^N) CaM. Components of each NMR sample are shown on each *left* vertical axis, and absence or presence of Ca^2+^ is displayed on the *top* of each spectrum. Sample names and Ca^2+^ conditions are color coded to match the spectra colors. CaM, calmodulin; NMR, nuclear magnetic resonance spectroscopy.

NMR spectroscopy is a powerful technique that can monitor binding events or conformational change of a protein or peptide in solution. For proteins that have been isotopically enriched with ^15^N, the x-y coordinates (resonance frequencies) of each ^1^H-^15^N cross-peak report information about the local environment. In a ^1^H-^15^N heteronuclear single quantum coherence spectra (HSQC), each cross-peaks correlates to an amino acid of the ^15^N enriched protein (amino acids that have side chains containing nitrogen also display cross-peaks). These HSQC spectra can be considered a fingerprint for the protein, and they are dependent on protein structure and/or complex lifetime. Within a series of controlled experiments (minimized changed in salt, temperature, and pH), changes in resonance frequencies that occur with the addition of a peptide or ligand are often referred to as chemical shift perturbations (CSPs). CSPs can arise from direct interaction or allosterically induced conformational change imparted to the isotopically enriched protein of interest (40). CaM-IQ motif: Figure 3 displays spectra of ^15^N CaM and ^15^N Na_V_1.5 CTD collected in the absence and presence of the Na_V_1.5 IQ motif peptide. Enlarged images of each spectrum are provided in Fig. S1. Our results are consistent with literature (24). CaM and the IQ motif interact in both the absence and presence of Ca^2+^ (Fig. 3, *A* and *B*), and these interactions are different (Fig. 3*C*). In the absence of Ca^2+^, addition of equal molar IQ motif peptide to ^15^N CaM caused many of the CaM-C domain cross-peaks to shift or disappear. (Fig. S2). In the presence of Ca^2+^, addition of the IQ motif had a different effect. CSPs in both the CaM-N and -C domains were observed (Fig. S3). CTD-IQ motif: The IQ motif peptide can also interact with the CTD. Addition of the IQ motif peptide altered the ^15^N CTD spectra in both the absence and presence of Ca^2+^ (Fig. 3, *D* and *E*). Comparing the CTD-IQ motif peptide spectra in the absence and presence of Ca^2+^ revealed that the CTD-IQ motif complex spectra are nearly identical (Fig. 3*F*). Previous efforts used fluorescence spectroscopy to characterize CTD-IQ motif binding. Data collected in the absence and presence of Ca^2+^ supported a shift in tryptophan fluorescence. This was interpreted as a Ca^2+^ dependent change in CTD-IQ motif affinity (9). To further understand this data, we repeated these fluorescence experiments using sub-stoichiometric additions of the IQ motif peptide to the CTD. We observed that the intrinsic fluorescence of tyrosine from the IQ motif peptide increased with each addition. At values beyond a 1:1 stoichiometric ratio (IQ motif peptide: CTD), the data appeared as a right shift in the tryptophan fluorescence signal. CaM-IGATE: Our previous report demonstrated ^15^N IGATE spectra were unaltered by the addition of apo CaM (confirmed by ITC) (28). In the presence of saturating Ca^2+^ (2 mM), the NMR spectra of ^15^N CaM displayed an abundance of CSPs upon the addition of IGATE peptide. These CSPs corresponded to amino acids located in both CaM-domains (Fig. S4). These data were consistent with our previous report where Ca^2+^ was required for CaM to engage the IGATE with both the -N and -C domains at two individual IGATE sites (28). CTD-IGATE: HSQC spectra of ^15^N CTD in the absence and presence of IGATE peptide displayed CSPs (Fig. S5). These data were consistent with electrostatic interactions between the Na_V_1.5 CTD glutamic acid and IGATE lysine amino acids (12). Mapping the CSPs onto the solution NMR structure (PDB ID 2KBI) supported a binding site interface comprised of CTD-helix I and IV. Identical CTD spectra and CSPs were observed irrespective of Ca^2+^. CTD-CaM: This construct (helices I-IV) did not display any CSPs upon the addition of CaM in either the absence or presence of Ca^2+^ (Fig. 3, *G*–*I*). Based on our previous MD simulations of an intact Na_V_1.5 alpha subunit (11, 12), the CTD construct used for this investigation (93 amino acids, E1773-S1865) was predicted to be sufficient for characterizing the binding interface and intermolecular forces of the CTD-IGATE and CTD-IQ motif interactions.

### 1:1:1 M ratio of CTD-IQ motif-CaM spectra are inconsistent with trimeric complex

**Figure 4:**
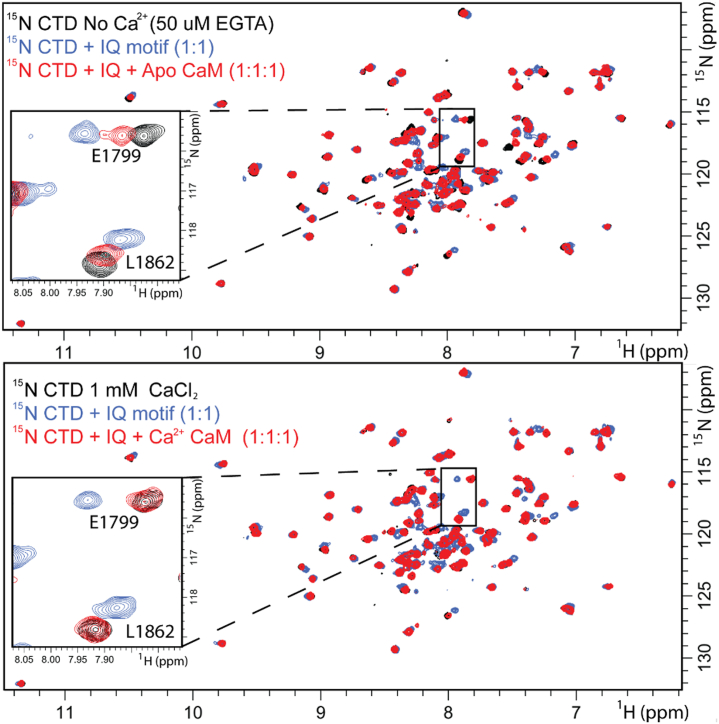
**Overlays of ^1^H-^15^N HSQC spectra of isotopically enriched ^15^N CTD collected in the: absence (*black*) and presence of IQ motif peptide (*blue*) or IQ motif peptide + CaM (*red*).** Data collected in the absence (*upper spectra*) and presence of Ca^2+^ (*lower spectra*). Assignments and resonance frequencies obtained during the course of ^15^N CTD: ^14^N CaM (1:1 stoichiometry) titrations with IQ motif peptide are provided in Fig. S7. Chemical shift perturbation color coded unto structure of CTD provided in Figure 5. CaM, calmodulin; CTD, C-terminal domain.

Based on the posited mechanistic model (27) and available crystallography data (25, 31, 32), we anticipated that the addition of CaM to the CTD-IQ motif complex would yield a third set of resonance frequencies consistent with formation of a transient complex. This would be visible in the data by displaying different resonance frequencies from the ^15^N CTD and ^15^N CTD-IQ complex spectra. Moreover, the line width of cross-peaks would broaden (and reduced amplitude) due to a slower tumbling rate of a larger complex. We also hypothesized that the addition of Ca^2+^ would restructure this trimeric complex, yielding a new variation of the trimeric NMR spectra. Assuming similarity between Na_V_1.2 (35) and Na_V_1.5 CaM-IQ data, we also anticipated that the CTD-IQ motif-CaM complex would be less stable in the presence of Ca^2+^ (*i.e.* contain multiple populations leading to a reduction in peak intensity and further enhancement to line broadening). Notably, the addition of CaM to the CTD-IQ motif sample did not yield these results. In the absence of Ca^2+^, stoichiometric addition of CaM to the ^15^N CTD-IQ motif sample yielded an NMR spectra with resonance frequencies in between those of CTD and the CTD-IQ motif complex (Fig. 4 top). Repeating this experiment in the presence of Ca^2+^ yielded a spectrum that was nearly identical to isolated ^15^N CTD (Fig. 4 bottom). Linewidths of the CTD-IQ motif complex were not altered by the presence of an equal molar ratio of CaM, irrespective of Ca^2+^ (Fig. S6).

**Figure 5:**
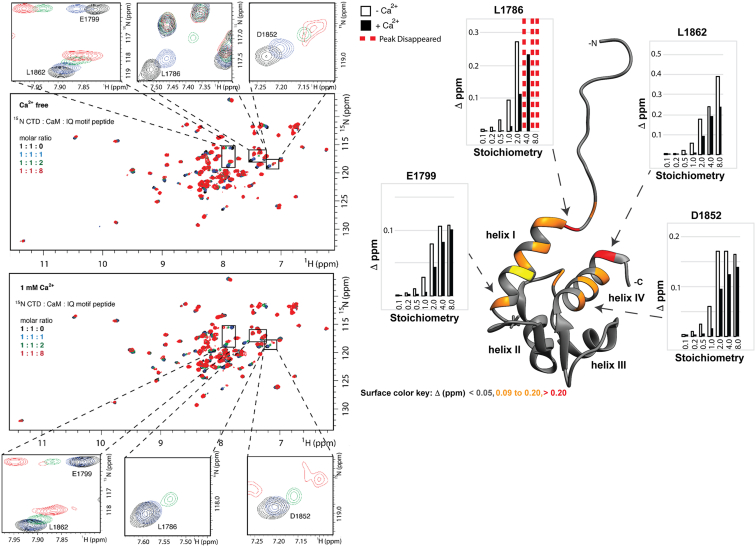
**Overlay ofHSQC NMR spectra of ^15^N CTD: ^14^N CaM with additions of IQ motif peptide in the absence (*top*) and presence of Ca^2+^ (*bottom*).** Insets for several amino acids that displayed prominent chemical shift perturbations (CSPs) are shown on the *left*. Prominent CSPs are color-coded on the surface of a ribbon rendering of the CTD structure (PDB ID: 2KBI). Plots of CSPs obtained during IQ motif titration in the absence (*open rectangles*) and presence of Ca^2+^ (*closed rectangles*) are shown on *right*. *Red dashes* indicate loss of cross-peak during titration. CaM, calmodulin; CSP, chemical shift perturbation; CTD, C-terminal domain; NMR, nuclear magnetic resonance spectroscopy.

To test the potential influence of stoichiometry on trimeric complex formation (CTD-IQ motif-CaM), we prepared samples of ^15^N CTD-CaM at a 1:1 M ratio and titrated in IQ motif peptide (Fig. 5). Analysis of the CSPs revealed that Na_V_1.5 CTD engaged the IQ motif peptide using helices I and IV. For all spectra (10% to 800% molar ratio of IQ motif peptide to ^15^N CTD + CaM samples) we observed resonance frequencies that could be reproduced by ^15^N CTD alone or ^15^N CTD-IQ motif peptide (Fig. S7). We also note that similar spectra were obtained at 1:1:8 (^15^N CTD: CaM: IQ motif peptide) in the absence and presence of Ca^2+^ (Fig. S8). These results indicate that a trimeric complex cannot be formed in solution from these components. Analysis of the structural data (PDB ID: 2L53 (24), CTD-IQ motif CSPs, and MD simulated CTD-IQ motif interaction (11)) supported that these CTD and CaM interactions engage the same binding site surface on the IQ motif. We concluded that CaM and the CTD compete for the IQ motif peptide in solution.

### Addition of Ca^2+^ enhanced CaM's ability to sequester IQ motif from CTD

To validate this finding, we repeated our 1:1:1 experiment in a controlled manner that excluded potential pipetting variations from our data. For this, we prepared 1.0 ml of equal molar ratios of ^15^N CTD, CaM, and IQ motif peptide (contained 30 μl of D_2_O for lock signal). This sample was split into two 500 μl samples, and Ca^2+^ was added to one. A small volume pH electrode was used to verify that the addition of Ca^2+^ to EGTA did not alter the sample pH (41). This methodology ensured that both samples were identical with the only difference being the absence or presence of Ca^2+^. The resulting spectral data were identical to our initial 1:1:1 experimental results (Fig. 4). In the absence of Ca^2+^, the 1:1:1 CTD-IQ motif-CaM spectra again displayed several cross-peaks that were in between that of the isolated ^15^N CTD and the ^15^N CTD-IQ motif complex. In the presence of Ca^2+^, the ^15^N CTD-IQ motif-CaM spectra were again identical to the isolated ^15^N CTD. To ensure the system had reached equilibrium, we recorded several NMR spectra of the 1:1:1 (^15^N CTD: IQ motif peptide: CaM) samples. One was collected immediately after the first experiment and others were taken at 1, 4, and 24 h later. The samples were stored at 4 °C in the NMR tube overnight, and then equilibrated to 298K for 15 min prior data collection. Identical spectra were obtained indicating that the protein system was at steady-state conditions. These experiments were repeated a third time with the IQ motif peptide being titrated into the ^15^N CTD-CaM samples. Plotting CSPs against IQ motif peptide additions illustrated that Ca^2+^ facilitated CaM's ability to sequester the IQ motif from the Na_V_1.5 CTD.

Ca^2+^ did not directly change the resonance frequencies of either sample. The addition of Ca^2+^ altered the amount of IQ motif required to induce CSPs in the ^15^N CTD spectra. Figures 5 and S7 display changes in resonance frequencies as the IQ motif peptide was added to the ^15^N CTD sample (also contained an equal molar ratio of CaM). On the left-hand side of Figure 5, the insets display shifts to amino acid resonance frequency with the addition of IQ motif peptide. On the right-hand side of the figure, these changes were quantified and are displayed as bar graphs. The open box values were obtained in the absence of Ca^2+^ and the closed boxes correspond to data collected in the presence of Ca^2+^. In the presence of Ca^2+^, more IQ motif peptide had to be added to the sample to elicit the CSPs observed in the absence of Ca^2+^.

### CTD used same binding surface to engage IQ motif or IGATE

Given the ability of NMR spectra to discern IQ motif preferential binding, we investigated the ability of the IQ motif to dislodge the CTD-IGATE interaction. Our recent modeling supported electrostatic interactions as the basis for the CTD-IGATE complex (12) and the CTD-IQ motif interaction was predicted to arise from hydrophobic packing (11). Given that the CTD-IGATE interaction dissociated in two out of four of our MD simulations and the CTD-IQ motif interaction was relatively stable across all four MD simulations, we hypothesized that the IQ motif could dislodge the CTD-IGATE interaction. Some CSPs were consistent with the hydrophobic CTD-IQ motif and electrostatic CTD-IGATE (Fig. S9). While distinct, most of these CSPs were small in magnitude. Overlaying the ^15^N CTD-IQ motif and ^15^N CTD-IGATE spectra revealed the CTD used the same helix I-IV surface area for interaction with both the IQ motif and IGATE peptides. This result was consistent with the available structural data and modeling predictions (10, 11, 12). Repeating the ^15^N CTD experiments in the presence of Ca^2+^ did not yield appreciable differences. As a result of the similarity between the CTD-IGATE and CTD-IQ motif spectra, we did not leverage these data as a competition experiment.

### CaM-N domain released Ca^2+^ more slowly in the presence of IGATE compared to IQ motif

**Figure 6:**
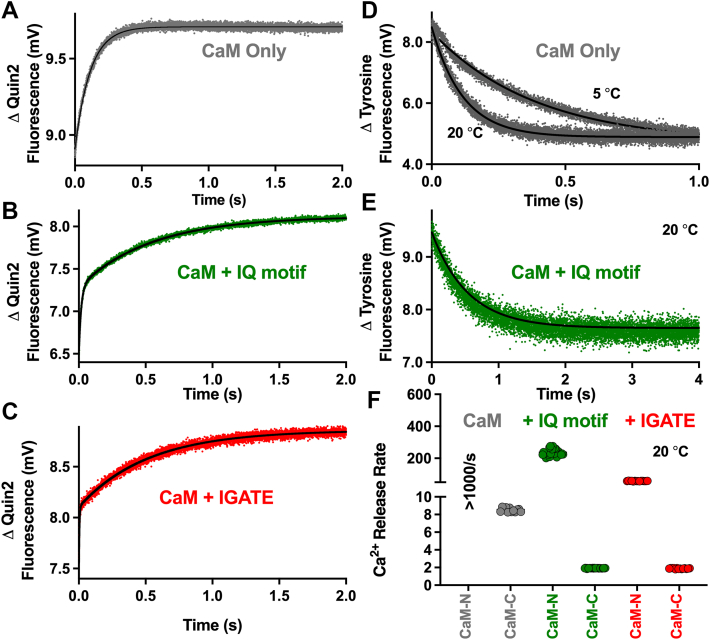
**Quantification of CaM domain kinetic rates.** Changes in Quin-2 fluorescence monitored over time to report on Ca^2+^ dissociation rates from (*A*) isolated CaM (*gray*), CaM in the presence of (*B*) IQ motif (*green*) or (*C*) IGATE peptide (*red*). Data were best fit to biexponential equations to obtain fast and slow Ca^2+^ release rates. *D and E*, assignment of slow Ca^2+^ release rates to CaM-C domain were derived from replicate experiments that monitored Tyr fluorescence (from CaM C-domain). *F*, summary of Ca^2+^ dissociation rates with individual replicates. Statistical difference between the CaM-IQ motif and CaM-IGATE rates were calculated using an unpaired student's *t* test with Welch's correction (*p* < 0.0001). CaM, calmodulin; IGATE, inactivation gate.

Having obtained the CTD mechanistic insights stated above, we next considered the CaM-mediated interactions (CaM-IQ motif and CaM-IGATE). Given solution NMR structures (CaM-Na_V_1.2 IQ motif) demonstrated that Ca^2+^ flipped the binding orientation of CaM, we pondered if the addition of Ca^2+^ could dislodge CaM from the Na_V_1.5 IQ motif in a meaningful timeframe that could allow transfer of the -N domain to the IGATE. For insight into this question, we turned to stopped-flow kinetics to quantify rates of Ca^2+^ dissociation. CaM was rapidly mixed with the fluorescent Ca^2+^ chelator Quin-2 as previously described (42). Quin-2 fluorescence was monitored as Ca^2+^ was removed from CaM in the absence or presence of peptides (Fig. 6).

At 20 °C, the fast component was too rapid to measure (occurred during mixing time of the stopped-flow apparatus, >1000 s^−1^) and the slow rate was 8.5 ± 0.2 s^−1^ (Fig. 6*A*). At 5 °C, isolated CaM data was best fit by two components with a fast rate of 636 ± 41 s^−1^, and slow rate of 2.7 ± 0.2 s^−1^ (5 °C Quin-2 data not shown). To measure Ca^2+^ dissociation rates from CaM in the presence of peptides, CaM was premixed with IGATE or IQ motif peptide at 1:3 M ratio in the presence of 30 μM Ca^2+^ to ensure saturation. These complexes were then rapidly mixed with Quin-2 at 20 °C. For the CaM-IQ motif complex, data were best fit by a two-component equation that contained fast (231 ± 15 s^−1^) and slow (1.92 ± 0.02 s^−1^) components. For the CaM-IGATE interaction, we also obtained Quin-2 data that was best fit by a two-component equation with fast (58 ± 1 s^−1^) and slow (1.88 ± 0.04 s^−1^) rates (Fig. 6, *B* and *C*). Similar to our previous work (43), we then sought to correlate each rate to a specific CaM domain. The intrinsic fluorescence of tyrosine can be used to monitor conformational change of the CaM-C domain over time (44). We repeated our experiments with EGTA in place of Quin-2 while monitoring tyrosine fluorescence. For isolated CaM, this yielded Ca^2+^ dissociation rates at 5 °C and 20 °C, of 2.6 ± 0.2 s^−1^ and 8.8 ± 0.2 s^−1^, respectively (Fig. 6*D*). This data confirmed the slower rates corresponded to the CaM-C domain. Tyrosine fluorescence was also used to verify the Ca^2+^ dissociation rate from the CaM-C domain in the presence of the IQ motif peptide (Fig. 6*E*). These data allowed us to correlate the slow component Ca^2+^ release to the CaM-C domain (1.94 ± 0.04/second), and the fast phase was attributed to the CaM-N domain (Fig. 6*F*). We note that tyrosine fluorescence was not used for CaM in the presence of the IGATE as the peptide contains multiple tyrosine residues (renders the signal ambiguous). Overall, the time required for CaM Ca^2+^ dissociation displayed both similarities and differences in the presence of IGATE and IQ motif peptides: (i) Both CaM peptide complexes released Ca^2+^ from the CaM-N domain more rapidly than the -C domain; and (ii) The CaM-N domain required more time to remove Ca^2+^ in the presence of the IGATE peptide compared to the IQ motif peptide (Fig. 6*F*).

## Discussion

### Intertwined mechanisms of channel gating and potential Ca^2+^ modification

**Figure 7:**
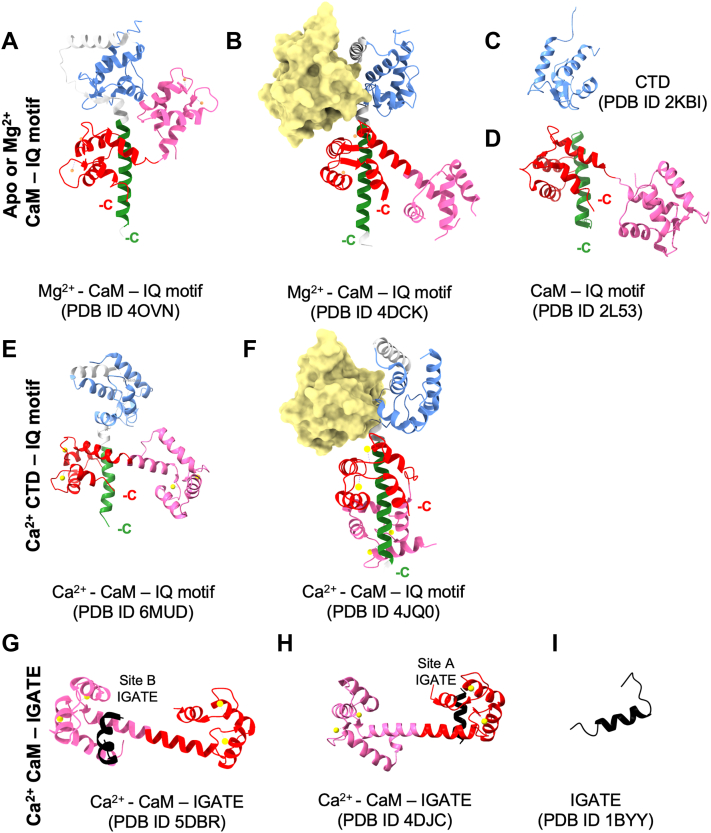
**Summary of empirical structural data of Na_V_1.5 components in the absence and presence of accessory proteins (CaM and fibroblast growth factors).** Ribbon renderings of: CTD (*blue*), IQ motif (*green*), IGATE (*black*), fibroblast growth factors (beige surface rendering), CaM-N domain (*pink*) and CaM-C domain (*red*). Data are grouped by protein or interaction: (*A* and *B*) CaM bound to CTD-IQ construct, (*C*) Isolated CTD, (*D*) CaM bound to IQ motif peptide, (*E*, *F*) Ca^2+^ CaM bound to CTD-IQ construct, (*G*, *H*) Ca^2+^ CaM bound to IGATE fragments, (*I*) Isolated IGATE. CaM, calmodulin; CTD, C-terminal domain; IGATE, inactivation gate.

Two intramolecular cytosolic CTD interactions can occur independent of Ca^2+^ (CTD-IQ motif, CTD-IGATE) (Fig. 1). Two CaM interactions are restructured or dependent on Ca^2+^ (CaM-IQ motif and CaM-IGATE, respectively) (Fig. 7). The interplay between these sets of CaM and CTD interactions has been the subject of extensive investigation (8, 10, 11, 12, 18, 19, 24, 25, 29, 38). The arrangement of these interactions into a cohesive molecular mechanism for channel modification requires clarification. Specifically, it is unclear if the CTD-IGATE and CTD-IQ motif interactions associate and dissociate with each action potential and if CaM is involved to modify Na_V_1.5 cytosolic structure. Our NMR and fluorescence data provided several important structural and kinetic insights for interpreting these protein-protein interactions. (i) CaM and the CTD could not simultaneously engage the IQ motif irrespective of Ca^2+^. (ii) The IQ motif and IGATE utilized the same binding site surface on the CTD. These interactions could not occur simultaneously irrespective of Ca^2+^. (iii) The presence of Ca^2+^ enhanced CaM's ability to sequester the IQ motif from the CTD. (iv) CaM-N domain Ca^2+^ dissociation required more time in the presence of the IGATE peptide relative to the IQ motif peptide. (v) The CaM-N domain does not form a stable complex with the IQ motif irrespective of Ca^2+^ (discussed below).

### CTD-IQ motif-CaM interactions in crystal compared to in solution

**Figure 8:**
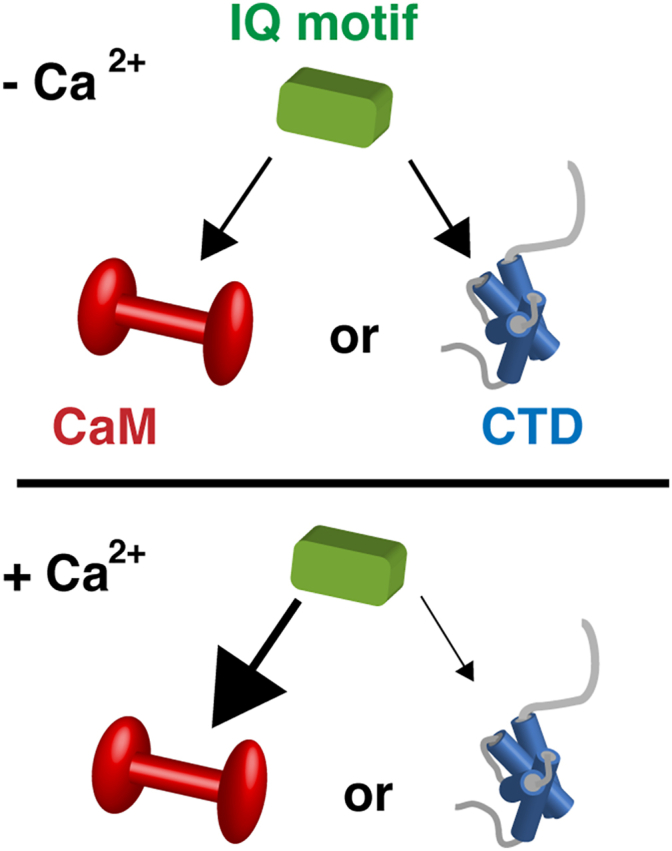
**Model illustrating IQ motif can interact with one binding partner at a time (CaM or CTD) irrespective of Ca^2+^.** IQ motif shown in *green*, CaM shown as *red dumbbell*, and CTD shown as *blue* bundle of four helixes. Arrow size indicates that CaMs ability to sequester the IQ motif was enhanced in the presence of Ca^2+^. CaM, calmodulin; CTD, C-terminal domain.

To date, one of the more comprehensive mechanisms posited for CaM-Na_V_1.5 modification contained the IQ motif of one channel contacting the CTD of a neighboring channel (27). Based on the arrangement of the proteins in the crystal lattice, we anticipated that a trimeric complex could be formed. Specifically, the N-terminal portion of the IQ motif would be engulfed by the CTD, and the C-terminal opposing face would be occupied by the CaM-C domain. We hypothesized that this complex could be restructured or undergo a significant reduction in complex lifetime upon the addition of Ca^2+^. Our NMR data were inconsistent with this aspect of the regulation model (27) and refuted our hypothesis. Instead, the data revealed that the IQ motif peptide could engage the CTD or CaM, but not both simultaneously (Fig. 8). To understand the mechanistic basis of the trimeric contacts observed in the 4DCK and 4OVN structures, we inspected the arrangement of proteins within the crystalline lattices. The crystallographic packing was consistent with open ended and cyclic domain swap configurations (Fig. S10).

### CaM IQ motif interactions provide a mechanism for dislodging IQ motif from CTD

Addition of CaM to the CTD-IQ motif complex removed the IQ motif from the CTD in both the absence and presence of Ca^2+^. CaM's ability to sequester the IQ motif from the CTD was enhanced in the presence of Ca^2+^. This finding is an opposite trend compared to Na_V_1.2 biophysical data (35). Close inspection of the CaM-Na_V_1.2 and CaM-Na_V_1.5 binding interfaces reveal that Na_V_1.2 contains a tyrosine (Na_V_1.2 Y1919) that served as an anchor for CaM interaction (34, 35). In Na_V_1.5, this tyrosine is replaced by a histidine. We speculate this substitution as a mechanism for the reduced CaM affinity observed for the Na_V_1.5 IQ motif peptide (25, 35). This sequence change may also underlie potential differences in Ca^2+^ CaM-C domain IQ motif binding orientation (24, 25). Additionally, histidine side chains often have pKa values around pHs that are physiological relevant, and therefore can serve as a pH sensor for enzymes and proteins (45). Given the location of this histidine at the CaM-IQ motif binding interface, further investigation will be required to understand the contribution to the CaM-IQ motif interaction.

### Ca^2+^kinetics hint at CaM domain specific roles within the context of an excitable cell

It is intriguing to speculate how differences in CaM Ca^2+^ release rates influence each complex lifetime within a human cardiomyocyte. Our stopped-flow kinetic data indicated that Ca^2+^ dissociation from the CaM-IQ motif complex can occur within the time scale of a resting human action potential (∼1 s). If the CaM-C domain is restructured by the addition of Ca^2+^ (parallel to anti-parallel similar to Na_V_1.2 CaM-IQ motif), this could provide a release mechanism for CaM to interact with the IGATE. Similarly, consideration of the CaM-IGATE Ca^2+^ release rates suggested this interaction could also occur on a beat to beat basis. Notably, the CaM Ca^2+^ release rates in the presence of IQ motif and IGATE were consistent with a paradigm where each CaM domain functions with a specific role in Ca^2+^ signal transduction. The faster Ca^2+^ release rates are consistent with the -N domain as a sensor that rapidly probe cytosolic [Ca^2+^], while the slower Ca^2+^ dissociation rates align with the -C domain as an anchor to a specific location.

### Structural and kinetic data support CaM-N domain as a Ca^2+^ sensor

The 4JQ0 crystal structure employed a longer IQ motif construct that extended to D1940; interestingly, the captured CaM-N domain binding site was comprised of R1913-S1925 (Fig. 7). The C terminal amino acids (F1928- D1940) were missing in the electron density data. This is consistent with the presence of multiple orientations (flexibility) and a lack of participation of these amino acids in the CaM-N domain interaction. When these residues were removed from C-terminus of the IQ motif (L1786-K1922), crystallography efforts (PDB ID 6MUD) captured the CaM-C domain engaged to the IQ motif construct while the CaM-N domain was oriented with the hydrophobic pocket exposed to the solvent. Based on our solution NMR and Ca^2+^ kinetic data, we posit that the CaM-N domain loosely interacts with the IQ motif in a transient manner that would allow it to continually sample the cytosolic [Ca^2+^]. In our stopped flow experiments, the CaM-N Ca^2+^ release rates were reduced in the presence of the IQ motif peptide (isolated CaM-N domain Ca^2+^ release is greater than 800 counts/second (43)). However, these rates were still faster than those observed in the presence of the IGATE peptide (Fig. 6). This was consistent with our NMR data where we observed well-defined CaM-N domain cross-peaks in the presence of IGATE, but not the IQ motif peptide (Fig. S11). Given the transient nature of the CaM-N domain interaction with the IQ motif, we posit that our previously identified site on IGATE (F1522-A1529, site B) could provide respite for an entropically burdened CaM-N domain hydrophobic core (exposed to the polar cytosol). Further work will be required to define the role of the CaM-N-Na_V_1.5 IGATE site B interaction.

### CaM-C domain as an arbitrator of the CTD-IQ motif and CTD-IGATE interactions

Our structural and kinetic data highlight several important features of the Na_V_-CaM interactions. The CaM-C domain can engage the IQ motif peptide in both the absence and presence of Ca^2+^, as well as the IGATE site A in the presence of Ca^2+^. Given CaM’s lack of ability to engage the IGATE in the absence of Ca^2+^, we speculate that the apo CaM IQ motif interaction is used to pre-localize CaM to the channel so it can rapidly access the channel for IGATE modification. When CaM is engaged to the IQ motif or IGATE, that particular site is unavailable for interaction with other parts of the channel. The complex lifetimes of these CaM interactions can aid in understanding the molecular mechanism of channel modification. Based on the nearly identical Ca^2+^ kinetics, we posit that the CaM-C domain IQ motif and CaM-C domain IGATE interactions are energetically balanced to serve as mediators. Specifically, CaM interaction can dictate the availability of the IGATE or IQ motif for interaction with the CTD, as well as the IGATE's availability to engage the channel latch receptor. We note that both the IGATE and IQ motif contain serine at the edge of the CaM-C domain binding sites (S1503 and S1904). Further work will be required to understand if and how additional factors such as posttranslational modification influence these interactions.

### Strengths and limitations of recombinant IQ motif and IGATE constructs used for interrogating CTD and CaM interaction

An intact Na_V_ alpha subunit requires a lipid bilayer for maintaining appropriate folding. Protein size and the lipid requirement currently render this incompatible with solution NMR. While isolated recombinant components can overcome these limitations, we caution that careful planning in construct design is essential for meaningful interpretion of the data. Below we describe our rational for each of our constructs that were used in this investigation. CTD, IQ motif, and IGATE constructs were designed based on available biophysical data with consideration of our recent modeling efforts. CTD (1773–1865): A solution NMR structure of the Na_V_1.5 CTD was determined for E1773-S1865 (10). This structure revealed four regions with high alpha helical content (helices I-IV) that were arranged into a globular structure with some resemblance to a CaM domain, albeit the typical acidic side chains for Ca^2+^ coordination were lacking in the loops. CryoEM structures (7), as well as our recent models of full-length Na_V_1.5 (alpha subunit in a lipid bilayer) (11), were consistent with these CTD structural features. Our model of the CTD-IQ motif interaction predicted the majority of the CTD-IQ motif binding interface was comprised of helix I-IV where the hydrophobic contacts were predominantly formed between Helix I and the IQ motif (F1912 sandwiched between F1791 and Y1795) (11).

#### IQ motif (E1901–S1925)

**Figure 9:**
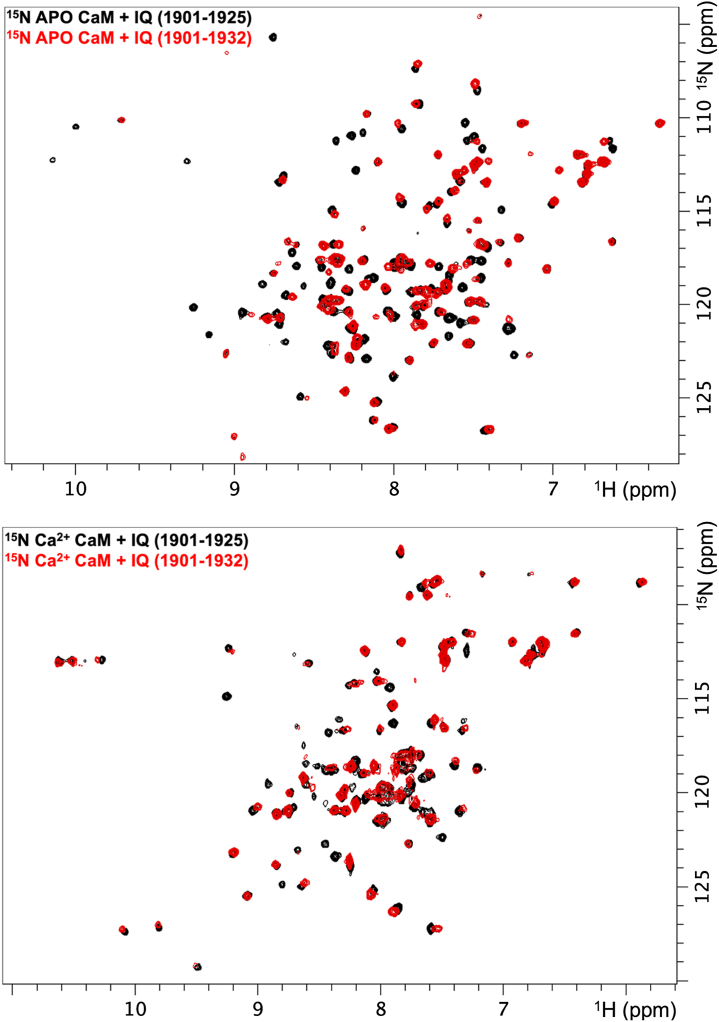
**^1^H ^15^N HSQC spectra of ^15^N CaM in presence of IQ motif (E1901-S1925) or (E1901-A1932).** Data was acquired in both the absence (*top*) and presence (*bottom*) of Ca^2+^ at the same temperature and pH as Figure 3, Figure 4, Figure 5. CaM, calmodulin.

For the IQ motif peptide construct, we selected E1901-S1925. This yielded well-defined HSQC spectra in the presence of apo and Ca^2+^ saturated CaM (Fig. 3, *A* and *B*). Based on the CaM-N domain interaction observed in PDB ID: 4JQ0, we also tested a C-terminal extended IQ motif construct (E1901-A1932). If the CaM-N domain engaged the C-terminally extended IQ motif with an appreciable complex lifetime, we expected to observe enhanced signal to noise in our data. This would arise from (i) improved isotropic tumbling rates that reduce coupling constants, as well as (ii) binding of the CaM-N domain that prelocalizes the CaM-C domain to increase overall complex lifetime. As shown in Figure 9, the additional C-terminal IQ motif amino acids reduced many of the cross-peak intensities. The remaining visible ^15^N CaM cross-peaks displayed nearly identical resonance frequencies to those observed in the presence of the shorter IQ motif peptide that was used for capturing the CaM-C domain IQ motif interaction. The spectra are consistent with a transient interaction between the CaM-N domain and the IQ motif that supports the CaM-N as a cytosolic Ca^2+^ sensor.

#### IGATE (Q1483–A1529)

In our previous work, we demonstrated that a C-terminally extended IGATE (Q1483-A1529) yielded an NMR spectrum consistent with a stable CaM-N domain interaction. Additionally, our crystal structure captured the CaM-N domain interaction with the IGATE F1522-A1529 (PDB ID 5DBR). ITC and NMR confirmed the importance of these contacts, as truncating these residues significantly weakened the overall CaM-IGATE affinity. Individual IGATE point mutations in each of the CaM binding sites yielded spectra with CSPs in a CaM domain specific manner. These were consistent with the CaM-N domain interacting with site B and the CaM-C domain with site A.

We note that the isolated protein constructs do not provide consideration for a fixed localization of the components as they would be within an intact channel. This has potential to influence kinetic association rates. Additionally, tertiary and quaternary structural arrangement has potential to sterically influence these interactions (impede or enhance). Further efforts aimed at tethering extended versions of these constructs to nano-discs, as well as incorporation into lipid vesicles for NMR solvent exchange measurements will be of benefit to expand upon our findings.

## Experimental procedures

### Sample production

Unlabeled and isotopically ^15^N enriched CaM, CTD, and IGATE protein (human sequence) were over expressed in BL21 (DE3) cells in LB or minimal media supplemented with ^15^N NHCl_4_. ^15^N ^13^C CaM was expressed in minimal media supplemented with both ^15^N NHCl_4_ and ^13^C glucose. Protein expression was induced with 1 mM IPTG. Cells were harvested by centrifugation and lysed by sonication. The CTD93 and IGATE constructs contained a cleavable His tag that was used for Ni^2+^ affinity purification. The IGATE construct contained a SUMO fusion protein to aid solubility and protection from proteolysis during protein expression. Both fusion protein and His tags were cleaved using HC3 protease. Ni^2+^ affinity was used to remove the His tag and SUMO -His Tagged fusion protein from the CTD93 and IGATE samples. The enriched CTD93 sample was purified using size exclusion chromatography. The IGATE construct was purified by reverse phase HPLC using a C4 Proto column with an acetonitrile gradient in the presence of 0.1% TFA. CaM did not contain a His tag and was purified using phenyl sepharose chromatography. Ca^2+^ was removed from CaM using 4 rounds of dialysis (2 × 4 L containing 5 mM EGTA followed by 2 × 4 L containing 50 μM EGTA). Concentrations were determined by UV vis spectroscopy using extinction coefficients (CaM ε = 3006 cm^−1^ M^−1^, IGATE ε = 4490 cm^−1^ M^−1^, CTD ε = 8480 cm^−1^ M^−1^ (9, 10, 28). IQ motif peptides were purchased from Innopep and hydrated in appropriate buffers for each experiment described below. IQ motif peptide concentration was calculated by weight. Purity, molecular weight, and stoichiometry of complex formation were validated by SDS gel electrophoresis (Fig. S12). Molecular weight of unlabeled and isotopically enriched proteins and peptides was verified by electrospray mass spectroscopy.

### NMR

^1^H-^15^N HSQC data were acquired and processed on 600 MHz and 800 MHz Bruker NMR spectrometers equipped with Cryogenic QCI or TCI probes using topspin 3.6 and 4.4. Water was suppressed using W5 Watergate pulse sequence. Samples were 50 to 100 μM protein in the presence of 50 mM Hepes, 100 mM KCl, 50 μM EGTA pH 7.4 with 1 μM DTT, 298K. Data were collected with 2048 points (direct ^1^H) x 256 (indirect ^15^N). Samples contained 3% D_2_O for a lock signal. Resonance frequencies were calibrated to H_2_O at 298 K. All spectra were processed using SSB = 2 without linear prediction. For experiments in the presence of Ca^2+^, samples were supplemented with 2 mM CaCl_2_. Resonance frequency assignments were transferred from Chagot (10) and Johnson lab data (verified by 3D HNCACB, CBCACONH, HNCA) (28, 46). Data were analyzed using SPARKY (https://www.cgl.ucsf.edu/home/sparky), POKY (https://sites.google.com/view/pokynmr) and Topspin 3.6 and 4.1 (https://www.bruker.com/en/products-and-solutions/mr/nmr-software/topspin.html) software. CSPs were calculated as described (43). Full width half max in Hz (Linewidths) were calculated using POKY analysis and fit was visually inspected using the view slice command. Topspin 4.1 deconvolution was used to resolve peaks that were overlapped when possible. Linewidths for resonances with significant overlap where peak shapes could not be clearly resolved or deconvoluted were omitted.

### Stopped flow

Data were acquired using an Applied Photo-physics stopped-flow instrument with a dead time of 1.4 msec at 5 °C or 20 °C (42, 47, 48). Samples were prepared using 3 μM CaM in the absence or presence of 9 μM peptide in buffer consisting of 10 mM Tris, 100 mM KCl, pH 7.4. Ca^2+^ dissociation rates were determined by rapidly mixing the CaM ± peptide + Ca^2+^ (30 μM) complex in buffer with 150 μM Quin-2 in buffer (λ_ex_ = 330 nm) or 10 mM EGTA in buffer (Tyrosine fluorescence λ_ex_ = 275 nm). Fluorescence of Quin-2 was monitored through a 510 broad band-pass interference filter (Oriel), while tyrosine fluorescence was monitored through a UV-transmitting black glass filter (UG1 from Oriel). Each Ca^2+^ dissociation rate represents an average of at least three traces with more than 10 replicates reported as the mean rate ± SD. The data were fit with single and double exponential equations to derive fast and slow rates (43).

### Structural analysis

PDB structures were visualized and compared using ChimeraX (https://www.cgl.ucsf.edu/chimerax). Figures were generated using ChimeraX, Adobe illustrator (https://www.adobe.com/products/illustrator.html), and GraphPad Prism (https://www.graphpad.com).

## Data availability

Data is available from the corresponding author (cn.johnson@chemistry.msstate.edu) upon request.

## Supporting information

This article contains supporting information (9, 10, 13, 16, 17, 19, 21, 24, 25, 26, 27, 28, 31, 35).

## Conflict of interest

The authors declare that they have no conflicts of interest with the contents of this article.
