# Supplementary material for: Molecular interactions of the NaV1.5 C-terminal domain: CaM sequestered the IQ motif from the CTD
Source: J Biol Chem. 2025 Oct 30;301(12):110871. doi: 10.1016/j.jbc.2025.110871 (PMC12682251; doi:10.1016/j.jbc.2025.110871)
Supplement: Supporting Figures and Table [file mmc1.pdf]

Table S1. Comprehensive summary of biophysical descriptions of Na_V: CTD, IQ motif, and CaM interactions.

Ref	Technique	Interaction	Construct (Na_V Sequence)	Affinity or Qualitative Statement	Comments
1	Gel shift	CaM + IQ	I1908-L1927	MLCK peptide out competes NaV for CaM	+ Ca^{2+}
2	Pull down	CaM + CTD-IQ	1870-1923	IQ/AA mutation = no detectable CaM interaction	1 mM Ca^{2+}
3	SDS gels of soluble and insoluble fractions	CaM + CTD-IQ	E1773-D1940	co-expression w/ CaM enhanced solubility	
	Size exclusion chromatography			CaM-NaV complex had similar retention time in absence and presence of Ca^{2+}	
4	Intrinsic tyrosine fluorescence	CaM-IQ	R1897-S1925	K_d = 0.16 μM	- Ca^{2+}
				K_d = 2.0 μM	+ Ca^{2+}
			IQ/AA mutation	K_d = 0.49 μM	- Ca^{2+}
				K_d = 3.4 μM	+ Ca^{2+}
5	Isothermal titration calorimetry	CTD-IQ	CTD = E1773-S1865<br>IQ = 1897-S1925	K_d = 27.5 μM	- Ca^{2+}
				K_d = 37.9 μM	+ Ca^{2+}
	Chemical shift perturbation			CSPs consistent w/ fast exchange -> moderate affinity	CTD helices I and IV used to interact with IQ motif
6	NMR	CaM-IQ	E1901-L1927	inter-molecular NOES (long complex lifetime) consistent with a high affinity	175 inter-molecular NOES<br>- Ca^{2+}
7	Isothermal titration calorimetry	CaM-IQ	1896-1924	K_d = 0.36 μM	- Ca^{2+}
		CaM-C-IQ		K_d = 0.34 μM	- Ca^{2+}
		CaM-N-IQ		no detectable binding	- Ca^{2+}
		CaM-N-IQ		K_d = 2.17 μM	+ Ca^{2+}
		CaM-C-IQ		K_d = 6.13 μM	+ Ca^{2+}
		CaM-C-CTD	1773-1925	K_d = 0.34 μM	- Ca^{2+}
		CaM-N-CTD		no detectable binding	- Ca^{2+}
		CaM-C and CaM-N-IQ	1896-1924	no detectable binding	+ Ca^{2+}
		CaM-CTD	1773-1892	no detectable binding	+ Ca^{2+}
		CaM-CTD	1773-1924	K_d = 2.06 μM	+ Ca^{2+}
		CaM-N-CTD		K_d = 2.53 μM	+ Ca^{2+}
		CaM-C-CTD		K_d = 7.63 μM	+ Ca^{2+}
		CaM-C and CaM-N CTD		no detectable binding	+ Ca^{2+}
		CaM-N and CaM-C-CTD		binding detected but not quantifiable	+ Ca^{2+}
8	Isothermal titration calorimetry	CaM + CTD-IQ	1773-1924	K_d = 1990 nM	+ Ca^{2+}
			1773-1940	K_d = 88 nM	- Ca^{2+}
			1773-1940	K_d = 132 nM	+ Ca^{2+}
			1773-1940 (A1924T)	K_d =497 nM	+ Ca^{2+}
			1773-1940 (A1924T)	K_d = 61 nM	- Ca^{2+}
9	Isothermal titration calorimetry	CaM + CTD-IQ	1776-1928	$K_d \approx$ 50 nM	- Ca^{2+}<br>binding curve not defined by data
10	Fluorescence anisotropy	CaM-IQ	rat Na_V1.2 K1901-K1927	K_d = 3.2 nM	- Ca^{2+}
		CaM-C-IQ		K_d = 11.6 nM	- Ca^{2+}
		CaM-N-IQ		K_d > 100 μM	- Ca^{2+}
		CaM-IQ		K_d = 85 nm	+ Ca^{2+}
		CaM-C-IQ		K_d = 386 nm	+ Ca^{2+}

		CaM-N-IQ		K_d > 100 μM	+ Ca^{2+}
11	Isothermal titration calorimetry	CaM + CTD-IQ	1773-1940	K_d = 99 nM	- Ca^{2+}
			1773-1940 (D1790G)	K_d = 128 nM	
			1773-1940 (Y1795C)	K_d = 283 nM	
			1773-1940 (Y1795H)	K_d = 103 nM	
			1773-1940 (E1901Q)	K_d = 243 nM	
			1773-1940 (Q1909R)	K_d = 32600 nM	
			1773-1940 (R1913H)	K_d = 2980 nM	
			1773-1940 (L1917K)	K_d = 75 nM	
			1773-1940 (IQ/AA)	no detectable binding	
12	Isothermal titration calorimetry	CaM-CTD	1773-1927	K_d = 1.02 μM	+ Ca^{2+}
			1773-1934	K_d = 0.26 μM	
			1773-1943	K_d = 0.4 μM	
			1773-1934 (FLF1926AAA)	K_d = 5 μM	
		CaM-C-CTD	1786-1925	K_d = 9 μM	
			1786-1925 (S1904L)	too weak to fit	
			1786-1925 (Q1909R)	K_d = 7 μM	
13	Isothermal titration calorimetry	CaM-CTD	E1773-D1940	K_d = 48 nM	- Ca^{2+}
			E1773-F1928	K_d = 39 nM	- Ca^{2+}
			E1773-D1940	K_d = 89 nM	- Ca^{2+}
			E1773-F1928	K_d = 1520 nM	+ Ca^{2+}
14	Isothermal titration calorimetry	CaM-IGATE	Q1483-A1529	K_d = 66 nM	+ Ca^{2+}
			Q1483-T1526	K_d = 0.83 μM	+ Ca^{2+}
			Q1483-D1523	K_d = 1.1 μM	+ Ca^{2+}
			Q1483-G1519	K_d = 4.6 μM	+ Ca^{2+}

References

1. Tan, H. L. *et al.* A calcium sensor in the sodium channel modulates cardiac excitability. 415, 442–447 (2002).
2. Deschenes, I. Isoform-Specific Modulation of Voltage-Gated Na^+ Channels by Calmodulin. *Circulation Research* 90, 49e–457 (2002).
3. Kim, J. *et al.* Calmodulin mediates Ca^{2+} sensitivity of sodium channels. *J. Biol. Chem.* 279, 45004–12 (2004).
4. Shah, V. N. *et al.* Calcium-dependent regulation of the voltage-gated sodium channel hH1: intrinsic and extrinsic sensors use a common molecular switch. *Proc. Nat. Acad. Sci.* 103, 3592–7 (2006).
5. Chagot, B., Potet, F., Balser, J. R. & Chazin, W. J. Solution NMR structure of the C-terminal EF-hand domain of human cardiac sodium channel $Na_V1.5$. *J. Biol. Chem.* 284, 6436–45 (2009).
6. Chagot, B. & Chazin, W. J. Solution NMR structure of Apo-calmodulin in complex with the IQ motif of human cardiac sodium channel $Na_V1.5$. *J. Mol. Biol.* 406, 106–19 (2011).
7. Sarhan, M. F., Tung, C.-C., Van Petegem, F. & Ahern, C. A. Crystallographic basis for calcium regulation of sodium channels. *Proc. Nat. Acad. Sci.* 109, 3558–63 (2012).
8. Wang, C. *et al.* Structural analyses of Ca^{2+} CaM interaction with Na_V channel C-termini reveal mechanisms of calcium-dependent regulation. *Nat. Comm.* 5, 4896 (2014).
9. Gabelli, S. B. *et al.* Regulation of the $Na_V1.5$ cytoplasmic domain by calmodulin. *Nat Comm.* 5, 5126 (2014).
10. Hovey, L. *et al.* Calcium triggers reversal of calmodulin on nested anti-parallel sites in the IQ motif of the neuronal voltage-dependent sodium channel $Na_V1.2$. *Biophys Chem* 224, 1–19 (2017).
11. Yan, H., Wang, C., Marx, S. O. & Pitt, G. S. Calmodulin limits pathogenic Na^+ channel persistent current. *The Journal of General Physiology* 1–17 (2017).
12. Gardill, B. R., Rivera-Acevedo, R. E., Tung, C.-C. & Van Petegem, F. Crystal structures of Ca^{2+} calmodulin bound to Na_V C-terminal regions suggest role for EF-hand domain in binding and inactivation. *Proceedings of the National Academy of Sciences* 201818618 (2019).
13. Yoder, J. B. *et al.* Ca^{2+} -dependent regulation of sodium channels $Na_V1.4$ and $Na_V1.5$ is controlled by the post-IQ motif. *Nat. Comm.* 10, 1–12 (2019).
14. Johnson, C. N., Potet, F., Thompson, M. K., Kroncke, B. M., Glazer, A. M., Voehler, M. W., Knollmann, B. C., George, A. L., and Chazin, W. J. A Mechanism of Calmodulin Modulation of the Human Cardiac Sodium Channel. *Structure*. 26, 683-694.e3 (2018).

Figure S1. Enlarged spectra of each panel shown in Figure 3.

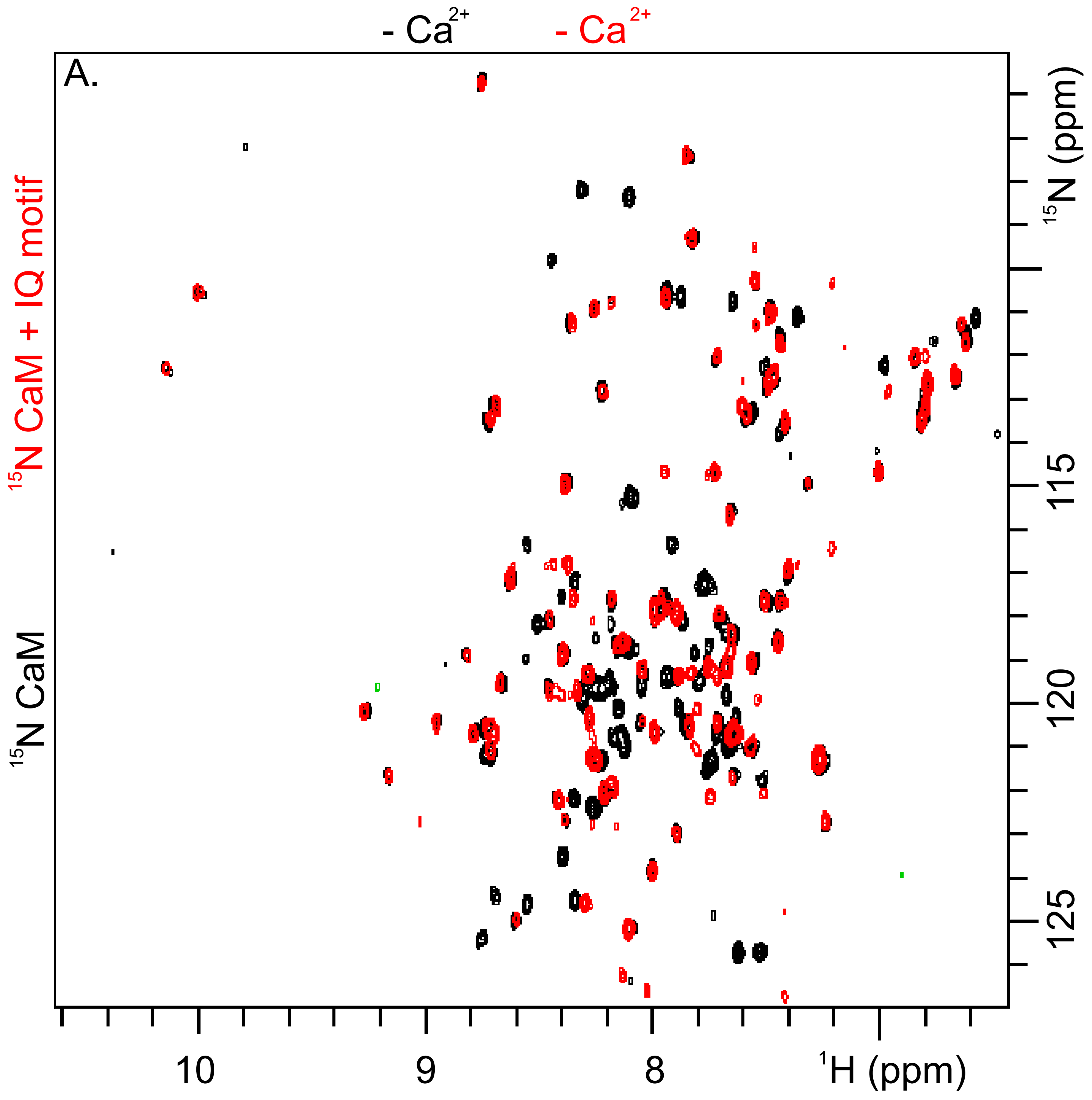

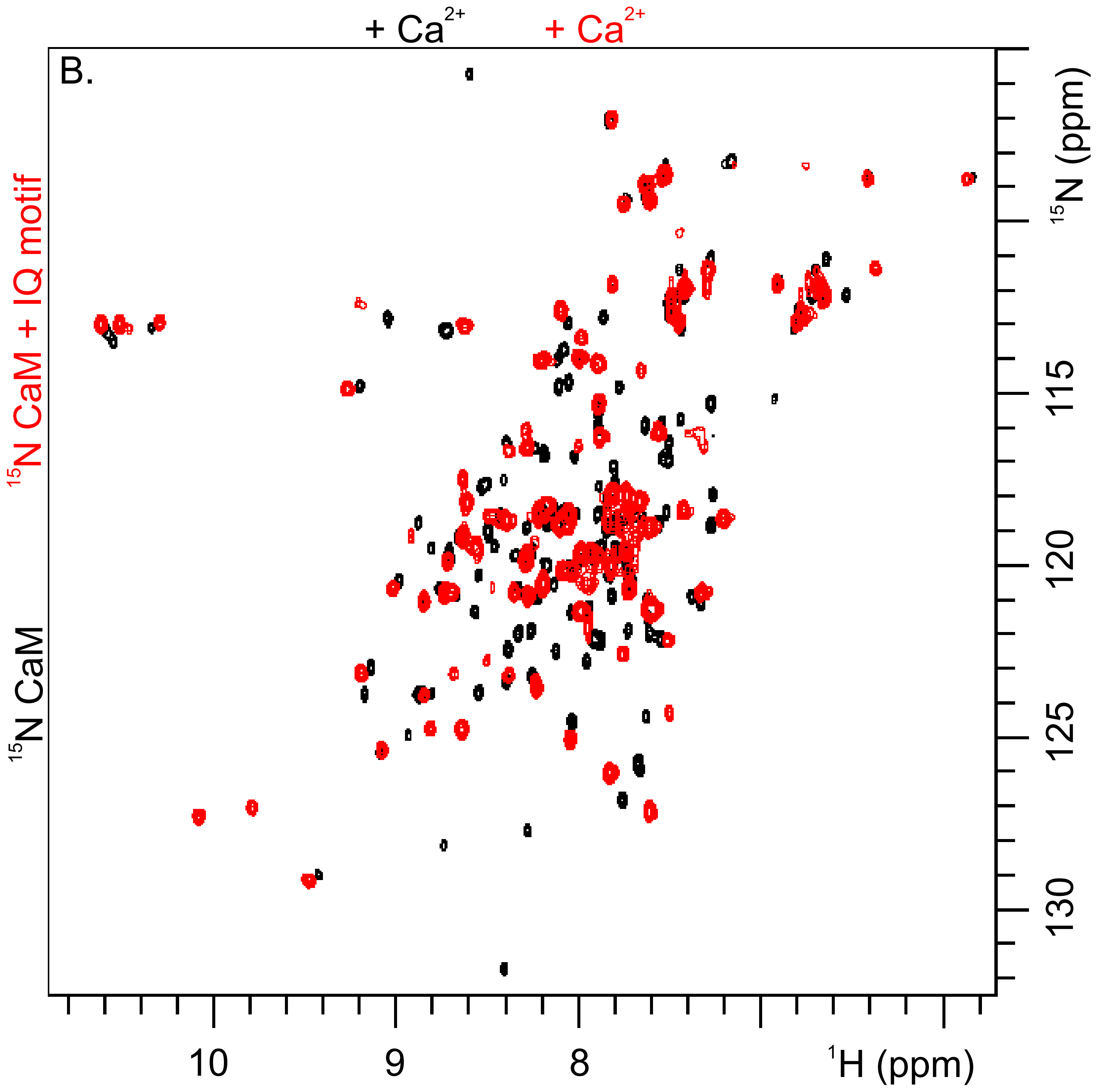
+ Ca2+
+ Ca2+
B.
15N CaM + IQ motif
15N CaM
15N (ppm)
115
120
125
130
10
9
8
1H (ppm)

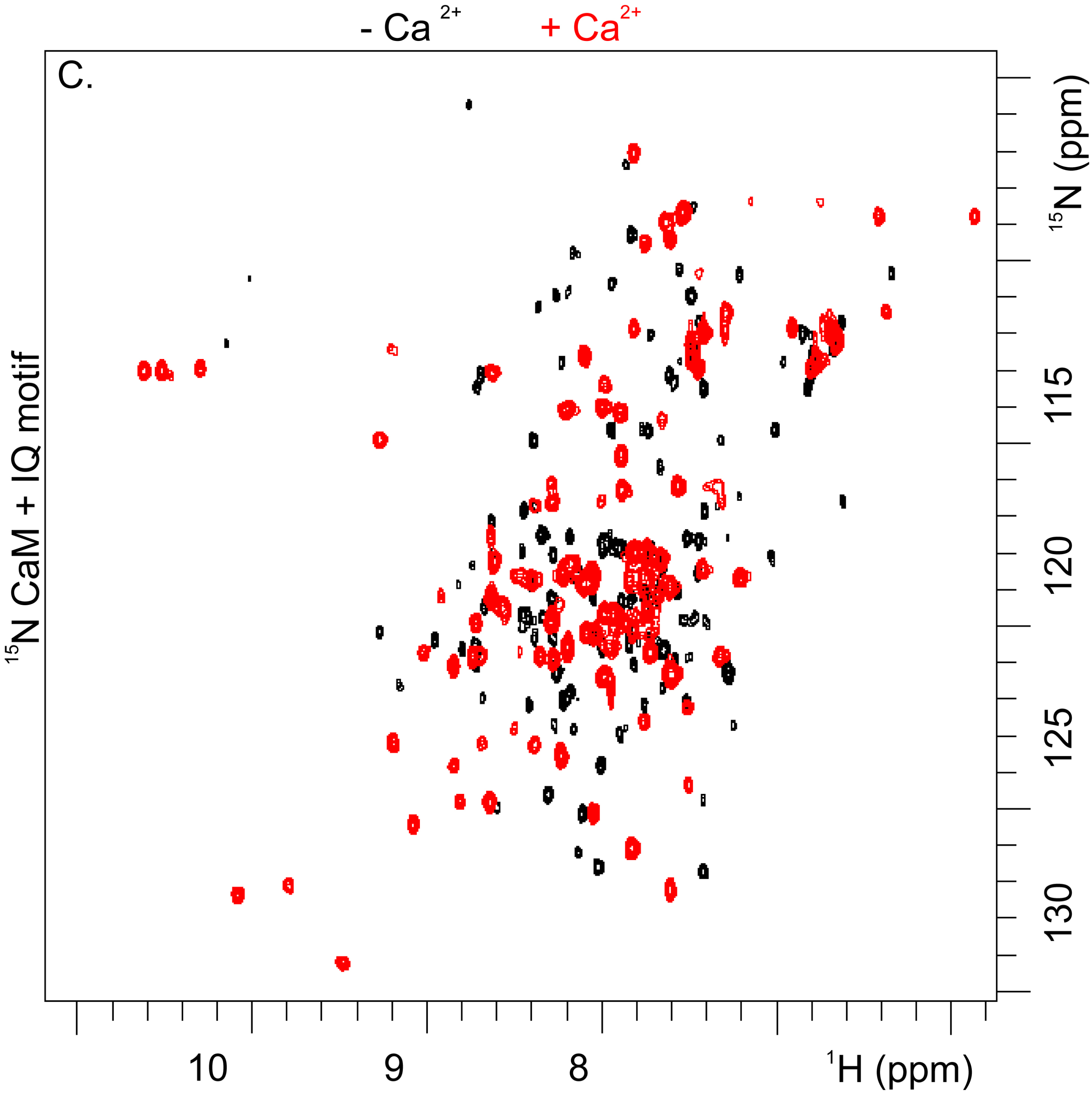

- Ca 2+
+ Ca2+
C.
15N CaM + IQ motif
15N (ppm)
115
120
125
130
10
9
8
1H (ppm)

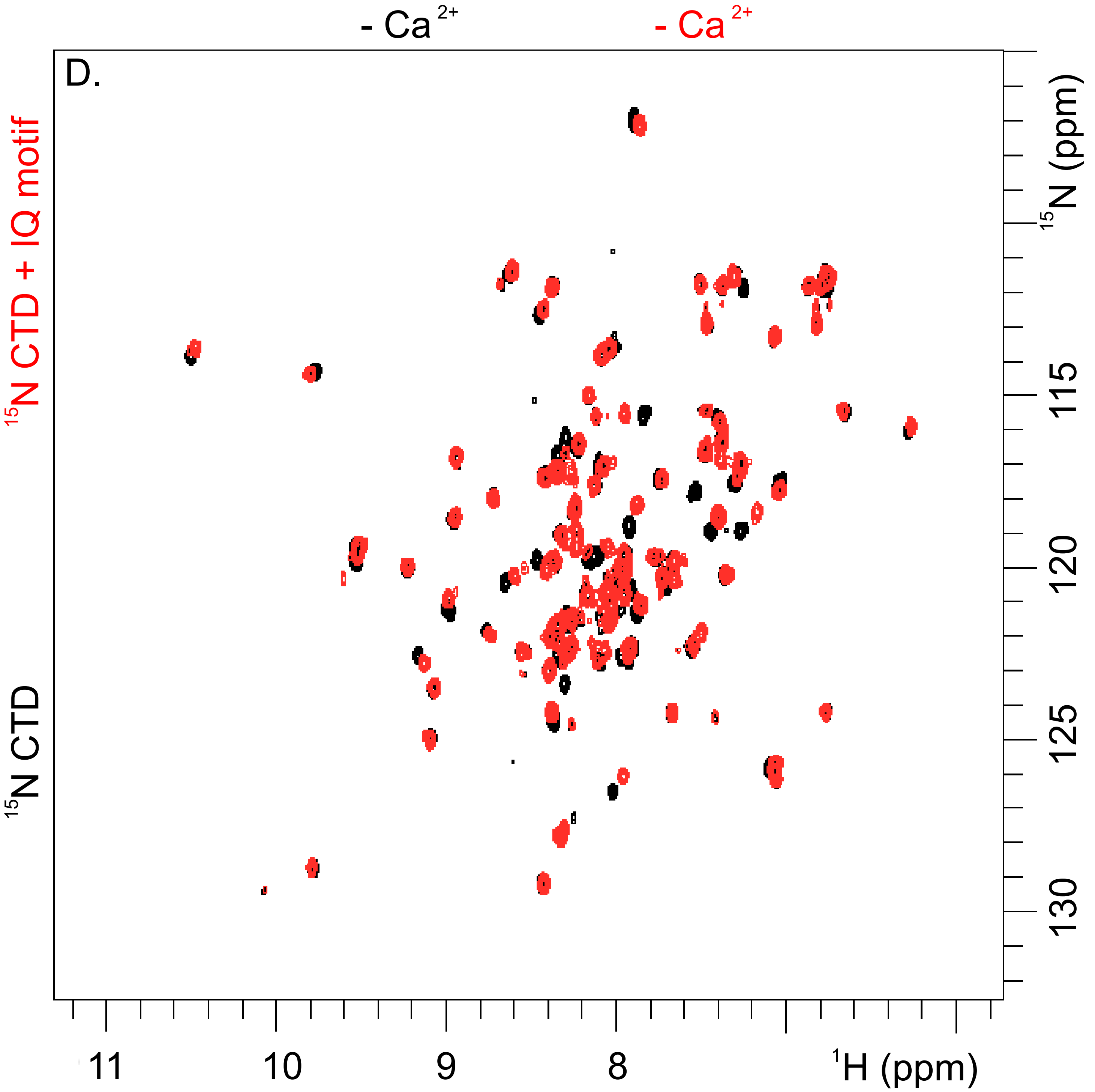
- Ca 2+
- Ca 2+
D.
15N CTD + IQ motif
15N CTD
15N (ppm)
115
120
125
130
11
10
9
8
1H (ppm)

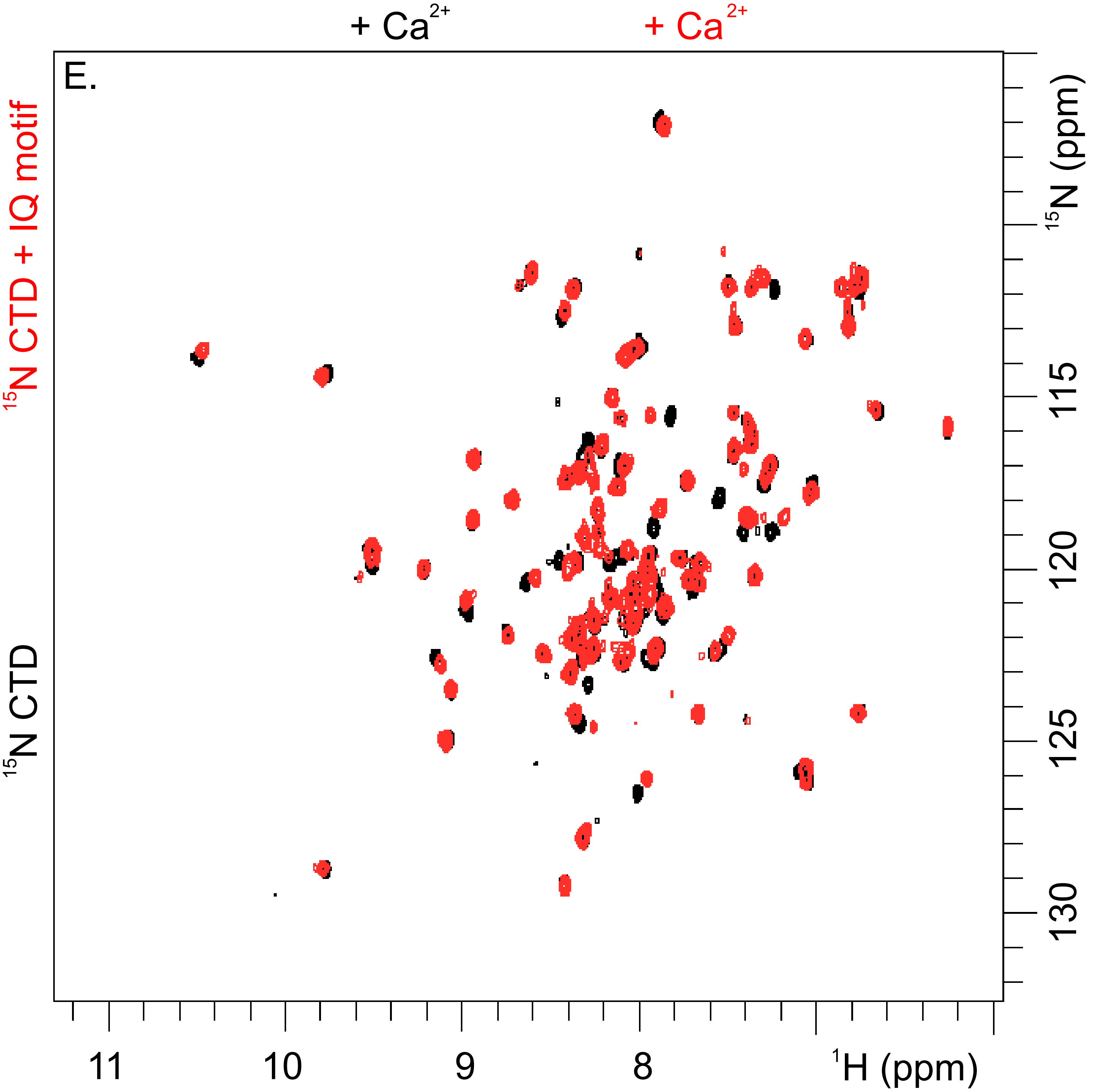

+ Ca2+
+ Ca2+
E.
15N CTD + IQ motif
15N CTD
15N (ppm)
115
120
125
130
11
10
9
8
1H (ppm)

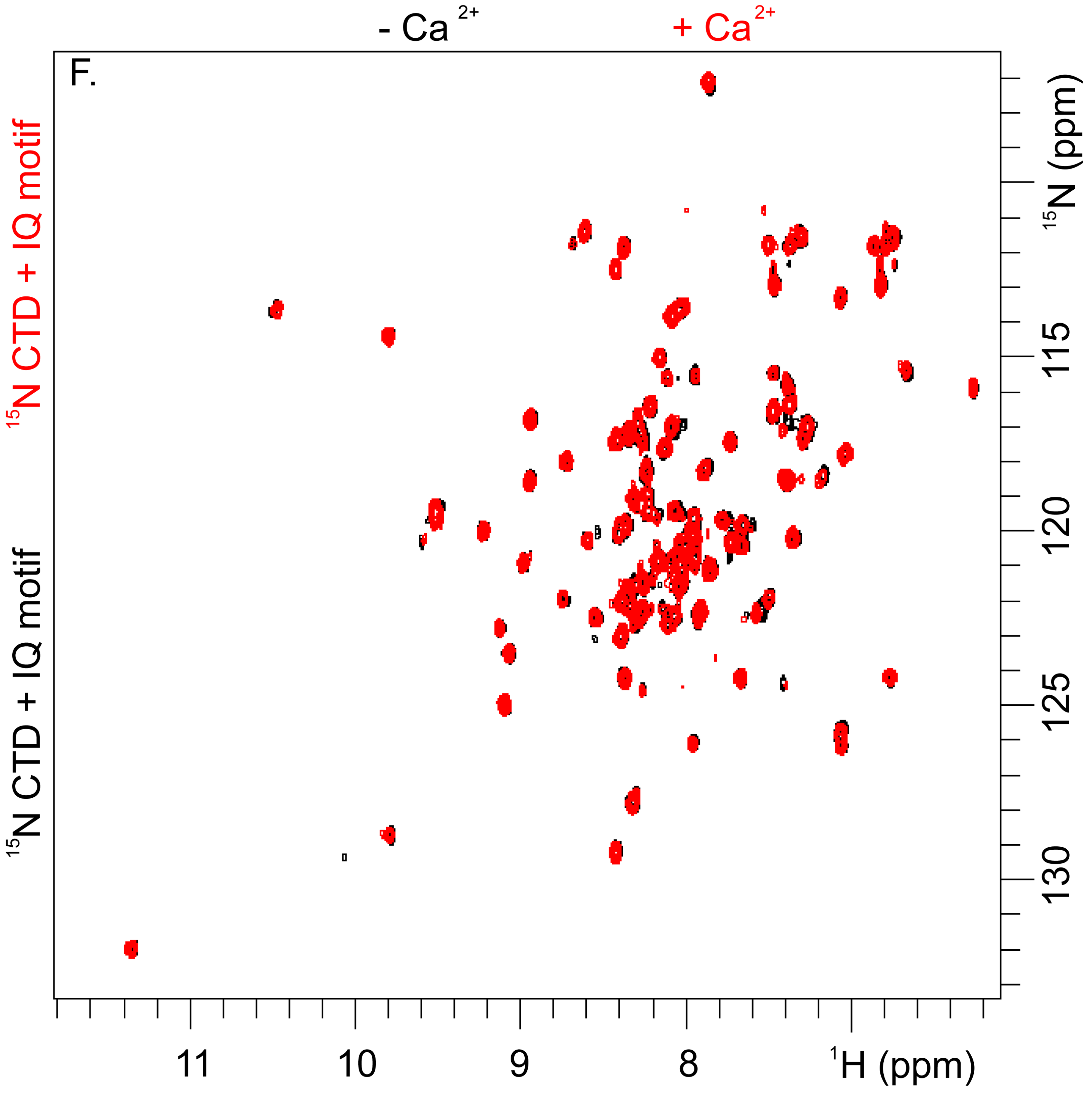

- Ca 2+
+ Ca2+
F.
15N CTD + IQ motif
15N CTD + IQ motif
15N (ppm)
115
120
125
130
11
10
9
8
1H (ppm)

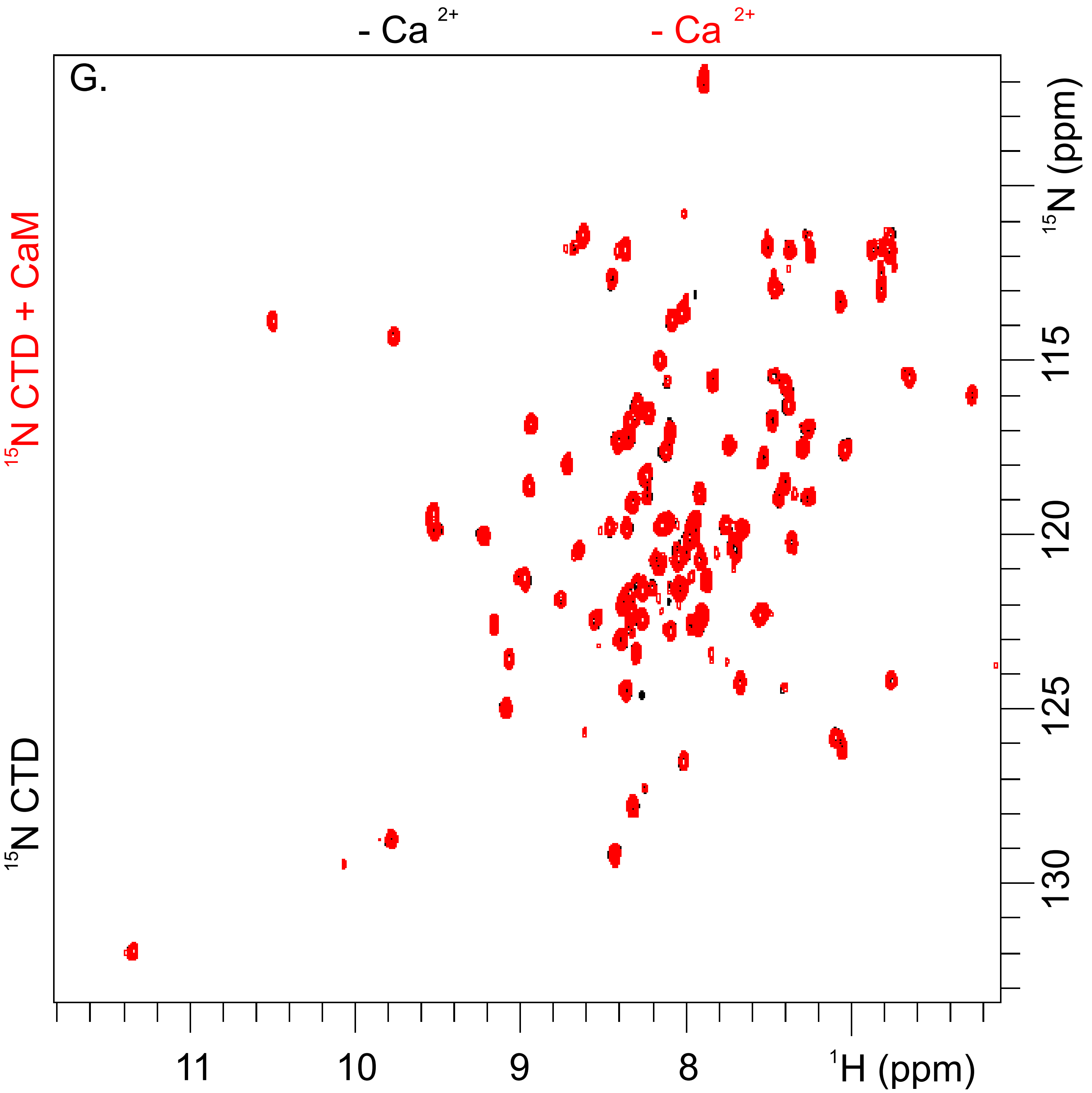

- Ca 2+
- Ca 2+
G.
15N CTD + CaM
15N CTD
15N (ppm)
115
120
125
130
11
10
9
8
1H (ppm)

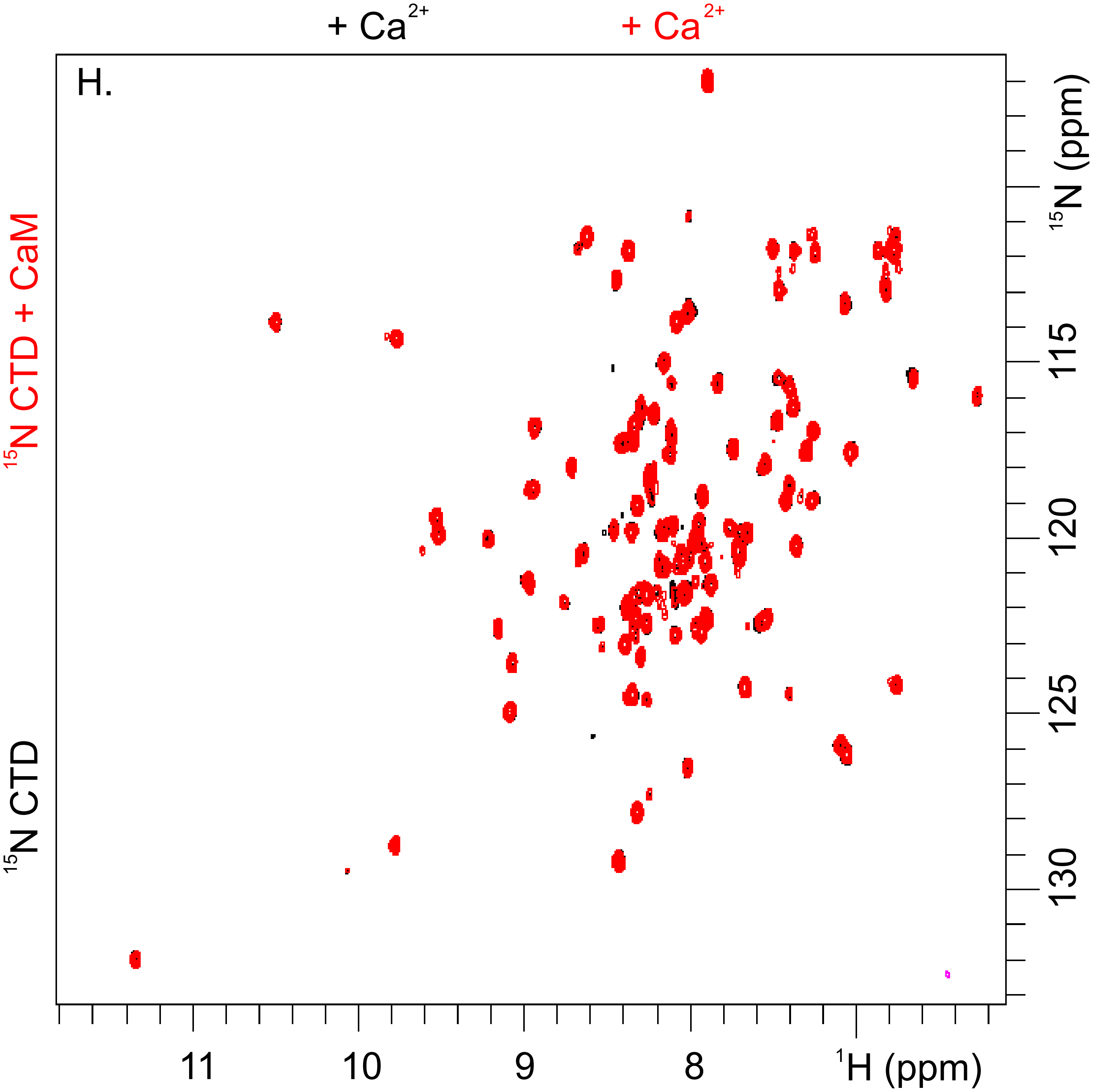

+ Ca2+
+ Ca2+
H.
15N CTD + CaM
15N CTD
15N (ppm)
115
120
125
130
11
10
9
8
1H (ppm)

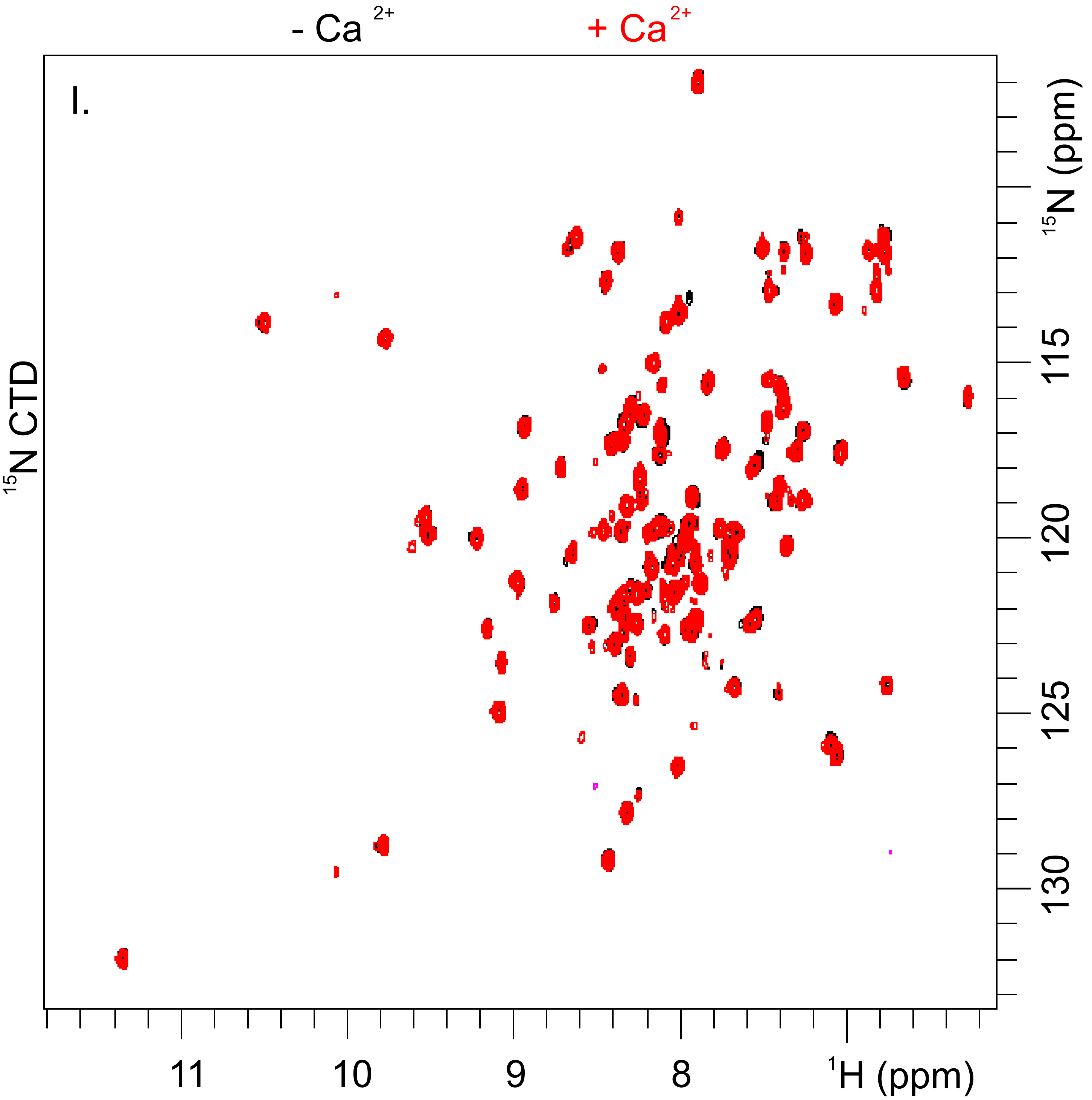
- Ca 2+
+ Ca 2+
I.
15N CTD
15N (ppm)
115
120
125
130
11
10
9
8
1H (ppm)

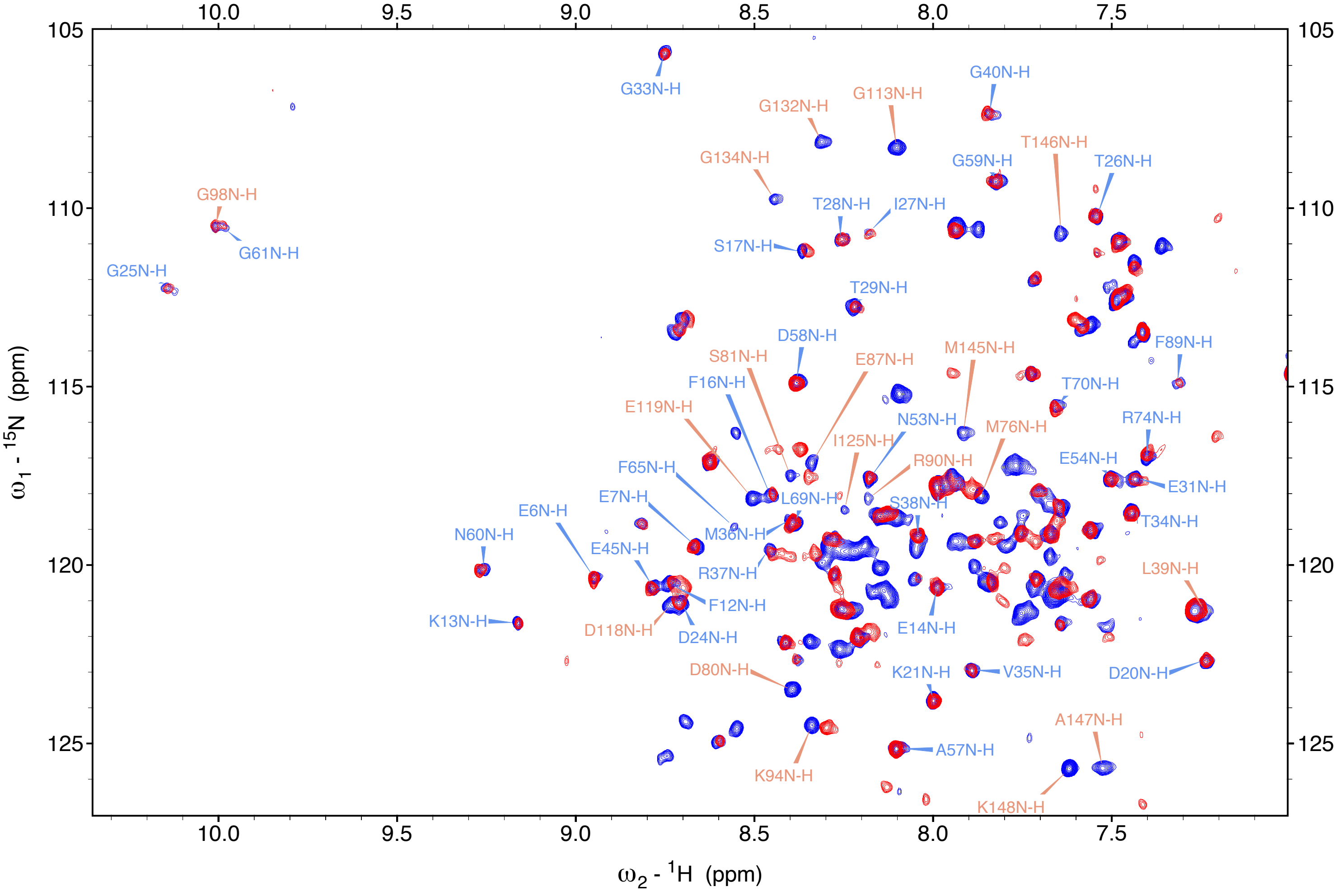

Figure S2. Overlay of HSQC ^{1}H-^{15}N NMR spectra of isotopically enriched ^{15}N CaM in the **absence** (blue) and **presence** (red) of IQ motif peptide (1901-1925) in the absence of Ca^{2+}. Select amino acid assignments are shown to demonstrate that only the CaM-C domain frequencies are altered by the IQ motif peptide. Labels are color coded **CaM-N** (amino acids 1-75) and **CaM-C** (amino acids 76-148).

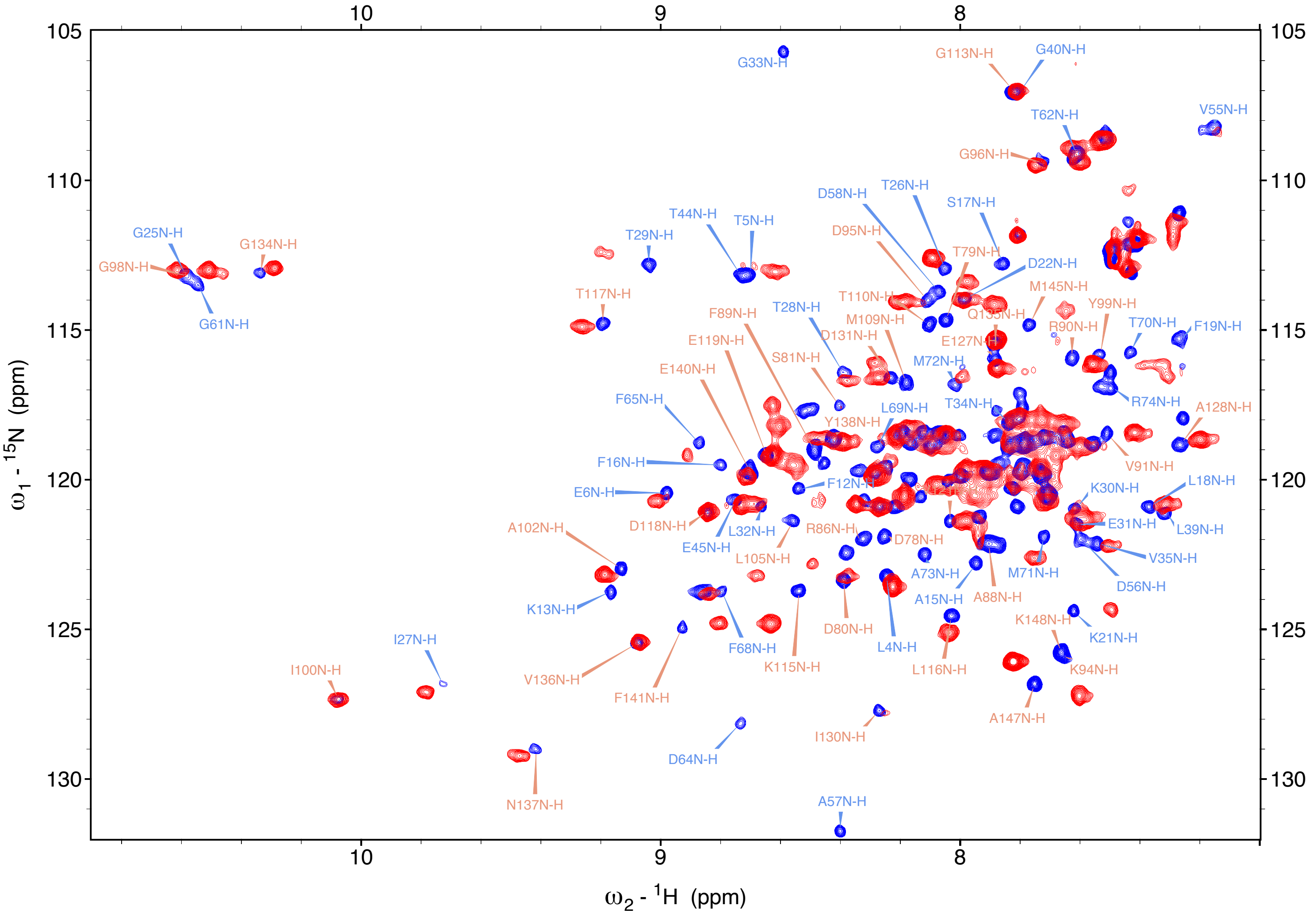

Figure S3. Both the CaM -N and -C domains interact with the IQ motif peptide in the presence of Ca^{2+}. Overlay of HSQC ^{1}H-^{15}N NMR spectra of isotopically enriched ^{15}N CaM in the **absence** (blue) and **presence** (red) of IQ motif peptide (E1901-S1925). Select amino acid assignments are shown to demonstrate that both the CaM-N and -C domain frequencies are altered by the IQ motif peptide. Labels are colored **CaM-N** (amino acids 1-75), **CaM-C** (amino acids 76-148).

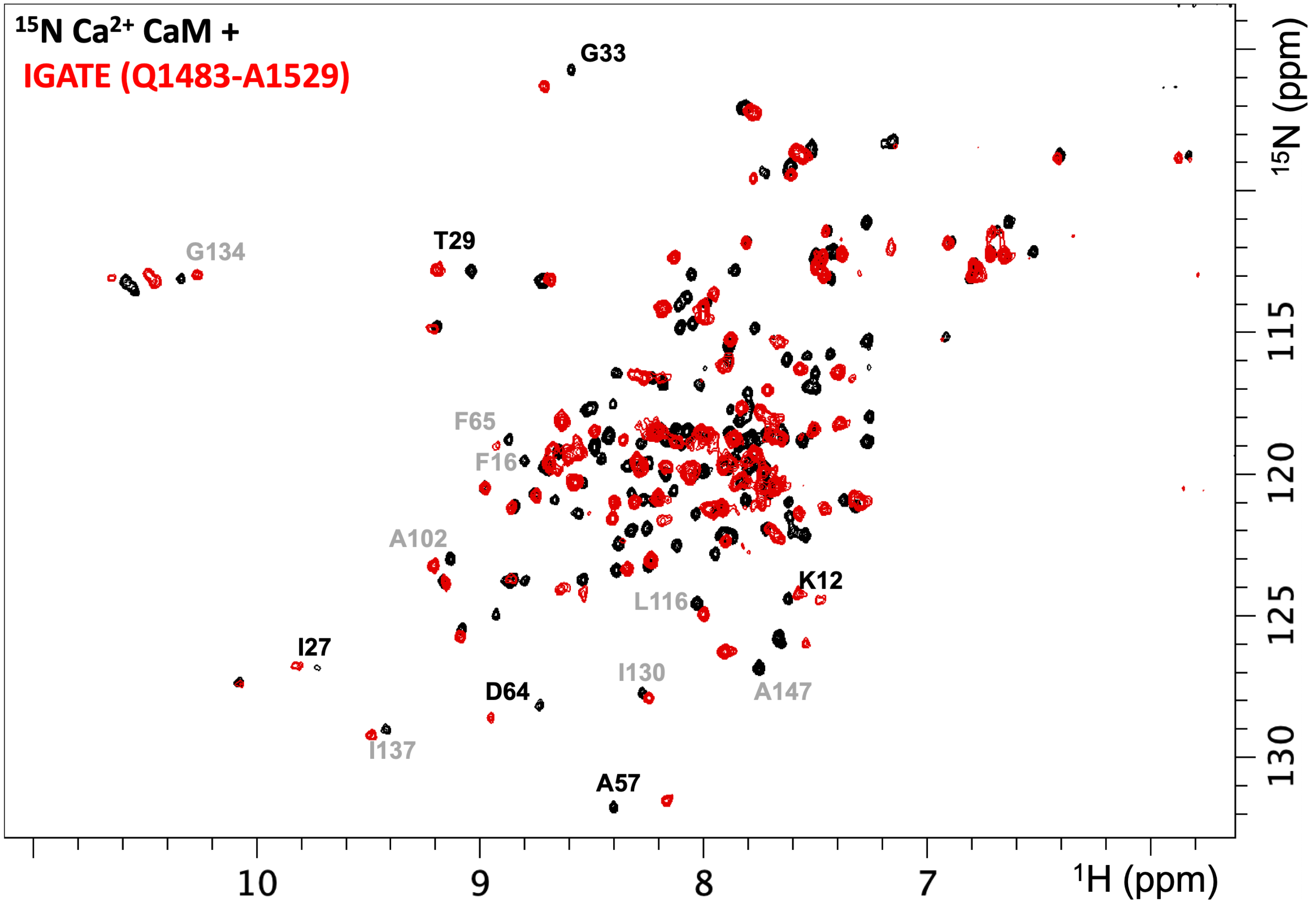

Figure S4. ^{1}H-^{15}N NMR spectra of isotopically enriched ^{15}N Ca^{2+} CaM in the absence (**black)** and presence (**red)** of IGATE peptide (Q1483-A1529). Several resonance frequency assignments are provided for both the CaM-N (black) and -C domain (grey) to demonstrate that both CaM domains engage the IGATE construct.

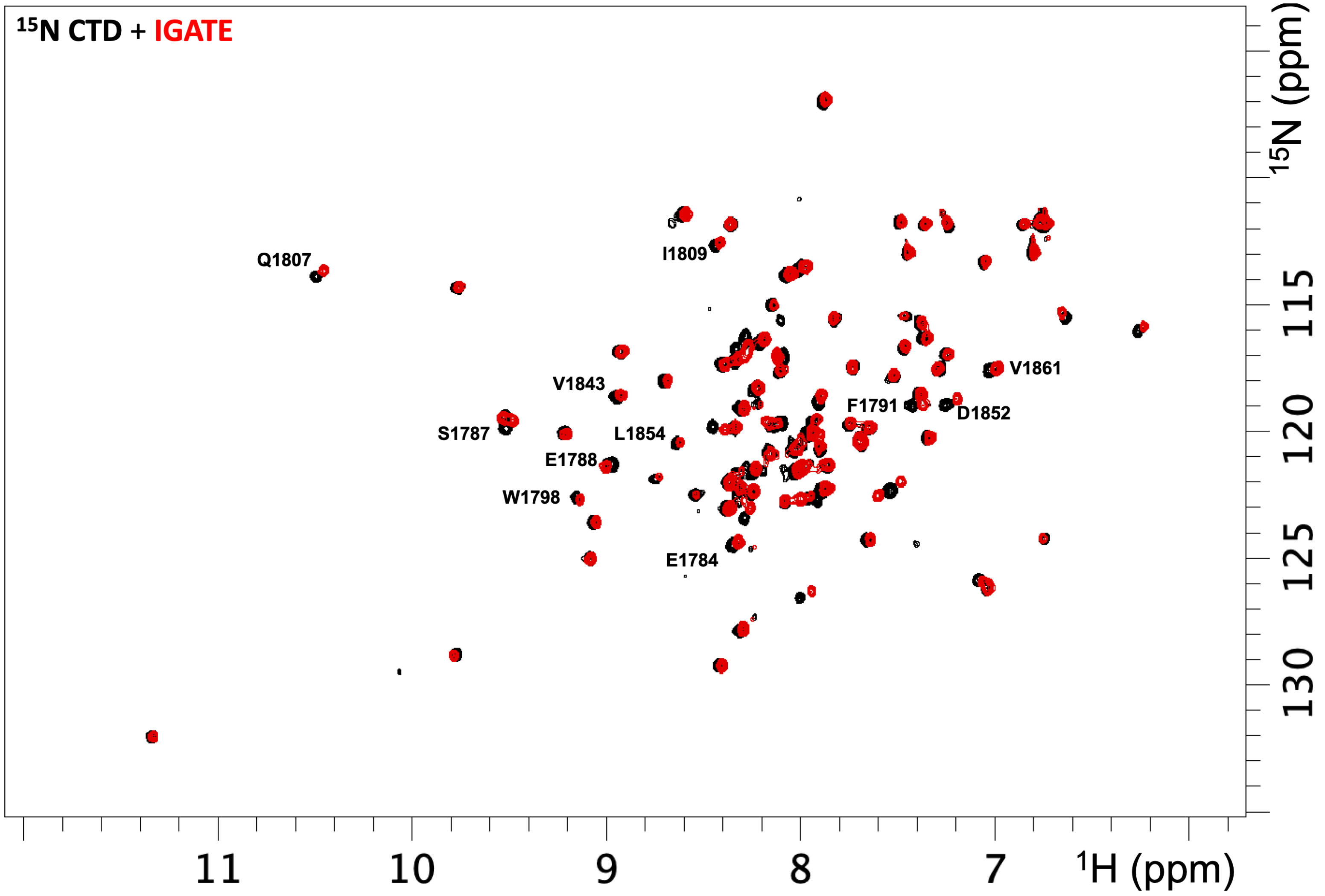

Figure S5. ^{1}H-^{15}N NMR spectra of isotopically enriched ^{15}N CTD in the absence (**black)** and presence (**red**) of IGATE peptide (Q1483-A1529). Altered resonance frequencies confirm CTD helix 1 and helix 4 as the IGATE binding interface in solution.

$-Ca^{2+}$	^{15}N CTD-IQ		^{15}N CTD + IQ + CaM			
RESID	^{15}N (Hz)	^{1}H (Hz)	^{15}N (Hz)	^{1}H (Hz)	^{15}N Δ	^{1}H Δ
E1773	21.3	28.1	22.1	26.1	0.8	-2.0
N1774	23.5	24.3	25.5	25.7	2.0	1.4
F1775	26.2	22.2	24.5	21.0	-1.7	-1.2
S1776	24.7	26.0	23.6	27.5	-1.1	1.5
V1777	20.2	19.8	20.7	19.2	0.5	-0.6
A1778	20.3	17.7	20.3	17.2	0.0	-0.5
T1779	20.7	19.6	20.9	19.3	0.2	-0.3
E1780	20.0	18.3	20.7	18.8	0.7	0.5
E1781	21.0	17.5	21.2	17.0	0.2	-0.5
S1782	22.2	19.7	22.9	21.1	0.7	1.4
T1783						
E1784	20.2	18.9	20.5	19.0	0.3	0.1
P1785						
L1786	22.1	22.0	22.5	25.4	0.4	3.4
S1787	20.8	26.0	22.2	26.7	1.4	0.7
E1788	24.3	19.0	25.3	22.8	1.0	3.8
D1789	23.5	17.0	21.7	17.3	-1.8	0.3
D1790						
F1791	21.8	24.8	24.6	21.8	2.8	-3.0
D1792						
M1793						
F1794						
Y1795	21.9	21.4	26.5	22.1	4.6	0.7
E1796	21.0	19.0	21.5	18.0	0.5	-1.0
I1797	23.5	29.6	24.8	29.6	1.3	0.0
W1798	21.6	19.8	22.6	21.9	1.0	2.1
E1799	23.3	22.1	22.7	23.2	-0.6	1.1
K1800	23.0	20.9	21.1	18.5	-1.9	-2.4
F1801	26.1	27.6	24.1	24.7	-2.0	-2.9
D1802						
P1803						
E1804	21.4	23.3	21.5	25.8	0.1	2.5
A1805	19.9	20.2	20.3	19.9	0.4	-0.3
T1806	21.5	24.0	21.8	25.6	0.3	1.6
Q1807	22.1	24.9	22.6	26.9	0.5	2.0
F1808	24.4	26.6	22.5	28.3	-1.9	1.7
I1809	22.8	21.6	24.0	23.7	1.2	2.1
E1810	25.6	22.4	25.7	23.4	0.1	1.0
Y1811	21.5	18.6	21.7	22.0	0.2	3.4
S1812	20.8	17.5	20.1	17.7	-0.7	0.2
V1813	23.9	25.1	23.5	23.8	-0.4	-1.3
L1814	22.3	20.4	20.7	19.5	-1.6	-0.9
S1815	22.2	22.9	21.9	23.5	-0.3	0.6
D1816						
F1817	21.2	22.2	21.5	23.0	0.3	0.8
A1818	20.9	19.9	22.1	21.5	1.2	1.6
D1819	20.1	18.5	20.4	20.9	0.3	2.4
A1820	21.1	19.5	20.9	20.1	-0.2	0.6
L1821						
S1822	22.8	25.7	26.3	25.4	3.5	-0.3
E1823	21.4	19.9	19.4	18.5	-2.0	-1.4
P1824						
L1825	22.4	28.0	23.1	27.8	0.7	-0.2
R1826	21.7	19.5	20.0	21.5	-1.7	2.0
I1827	22.3	29.2	22.4	29.5	0.1	0.3
A1828	20.9	18.9	20.3	19.3	-0.6	0.4
K1829	23.7	23.2	22.2	25.5	-1.5	2.3
P1830						
N1831						
Q1832	29.2	27.6	28.4	25.1	-0.8	-2.5
I1833	26.9	28.2	28.9	29.2	2.0	1.0
S1834	21.9	29.0	21.4	31.0	-0.5	2.0
L1835	24.0	27.1	25.3	26.9	1.3	-0.2
I1836						
N1837	20.5	19.2	20.8	21.1	0.3	1.9
M1838	20.4	18.2	21.5	18.3	1.1	0.1
D1839	21.1	23.5	22.5	25.0	1.4	1.5
L1840	22.2	31.5	20.7	29.4	-1.5	-2.1
P1841						
M1842	22.9	22.6	23.6	23.1	0.7	0.5
V1843	21.6	24.3	22.1	25.6	0.5	1.3
S1844	23.3	24.0	23.0	25.3	-0.3	1.3
G1845	24.9	32.9	28.7	31.5	3.8	-1.4
D1846	20.9	20.7	22.1	21.2	1.2	0.5
R1847	20.3	24.0	20.5	26.0	0.2	2.0
I1848	21.9	26.3	20.9	27.6	-1.0	1.3
H1849	21.8	21.9	20.8	24.2	-1.0	2.3
C1850						
M1851						
D1852	22.6	27.7	22.0	26.5	-0.6	-1.2
I1853						
L1854	22.4	24.2	23.9	25.1	1.5	0.9
F1855	22.3	24.4	22.7	24.9	0.4	0.5
A1856	23.0	23.6	22.2	23.7	-0.8	0.1
F1857	23.1	24.3	23.7	24.5	0.6	0.2
T1858						
K1859	20.2	24.0	20.7	26.1	0.5	2.1
R1860	18.9	18.7	20.9	18.5	2.0	-0.2
V1861	22.9	24.8	22.0	24.0	-0.9	-0.8
L1862	22.3	19.5	23.2	21.9	0.9	2.4
G1863	21.2	19.3	22.1	21.5	0.9	2.2
E1864	19.8	19.1	19.9	19.5	0.1	0.4
S1865	19.3	19.0	19.8	18.2	0.5	-0.8

$+Ca^{2+}$	^{15}N CTD-IQ		^{15}N CTD + IQ + CaM			
	^{15}N (Hz)	^{1}H (Hz)	^{15}N (Hz)	^{1}H (Hz)	^{15}N Δ	^{1}H Δ
E1773	23.1	27.3	21.8	32.3	-1.3	5.0
N1774	29.3	29.6	32.2	29.2	2.9	-0.4
F1775	34.4	33.3	34.6	29.5	0.2	-3.8
S1776	24.1	29.6	22.7	27.2	-1.4	-2.4
V1777	21.4	23.0	21.4	20.6	0.0	-2.4
A1778	20.9	20.0	20.3	17.6	-0.6	-2.4
T1779	21.1	22.6	20.2	19.4	-0.9	-3.2
E1780	21.0	22.3	20.9	19.4	-0.1	-2.9
E1781	22.3	20.5	20.2	17.6	-2.1	-2.9
S1782	24.7	25.4	23.6	25.6	-1.1	0.2
T1783	23.4	25.0	23.0	22.2	-0.4	-2.8
E1784	20.7	22.5	20.6	19.6	-0.1	-2.9
P1785						
L1786	21.7	23.7	22.5	26.3	0.8	2.6
S1787	20.5	30.4	21.8	31.2	1.3	0.8
E1788	24.5	25.1	26.0	24.7	1.5	-0.4
D1789	24.9	22.3	25.0	20.4	0.1	-1.9
D1790	22.1	22.2	27.4	28.1	5.3	5.9
F1791	24.8	27.1	22.3	22.4	-2.5	-4.7
D1792						
M1793						
F1794						
Y1795	22.4	24.5	26.4	24.4	4.0	-0.1
E1796						
I1797	25.5	29.5	26.1	27.9	0.6	-1.6
W1798	21.7	20.5	22.8	21.1	1.1	0.6
E1799	22.4	23.6	22.8	29.5	0.4	5.9
K1800						
F1801						
D1802						
P1803						
E1804	21.7	25.4	21.5	25.0	-0.2	-0.4
A1805	22.7	22.3	22.1	21.2	-0.6	-1.1
T1806	22.6	29.2	21.2	30.0	-1.4	0.8
Q1807	22.7	31.0	21.6	28.9	-1.1	-2.1
F1808	25.3	26.9	22.8	27.3	-2.5	0.4
I1809	26.8	25.9	26.3	25.9	-0.5	0.0
E1810	26.3	24.0	26.5	25.0	0.2	1.0
Y1811	21.6	22.7	21.6	20.6	0.0	-2.1
S1812	21.7	21.7	21.6	20.2	-0.1	-1.5
V1813	27.0	28.7	28.5	26.9	1.5	-1.8
L1814	23.1	25.9	22.3	20.8	-0.8	-5.1
S1815	21.7	23.4	19.5	20.2	-2.2	-3.2
D1816						
F1817	22.8	22.7	23.8	24.6	1.0	1.9
A1818	22.0	25.8	22.1	22.2	0.1	-3.6
D1819	22.0	22.5	21.6	23.4	-0.4	0.9
A1820	22.6	22.9	23.2	21.9	0.6	-1.0
L1821	23.6	23.7	22.3	21.2	-1.3	-2.5
S1822	25.3	31.6	24.5	28.8	-0.8	-2.8
E1823	21.7	22.8	23.0	22.6	1.3	-0.2
P1824						
L1825						
R1826	23.2	22.0	22.4	25.9	-0.8	3.9
I1827	22.5	45.3	22.5	39.1	0.0	-6.2
A1828	21.0	23.2	20.9	20.8	-0.1	-2.4
K1829	23.6	27.7	23.2	25.9	-0.4	-1.8
P1830						
N1831						
Q1832	25.9	25.1	25.2	23.0	-0.7	-2.1
I1833	29.3	28.2	26.7	23.2	-2.6	-5.0
S1834	21.9	30.1	20.9	31.7	-1.0	1.6
L1835	22.0	23.4	23.6	24.8	1.6	1.4
I1836						
N1837	21.1	21.4	22.1	23.2	1.0	1.8
M1838	22.0	20.6	22.3	20.4	0.3	-0.2
D1839	26.5	30.1	25.5	29.3	-1.0	-0.8
L1840	24.2	33.1	21.9	31.5	-2.3	-1.6
P1841						
M1842	23.4	24.4	23.0	22.0	-0.4	-2.4
V1843	21.6	26.9	22.4	25.4	0.8	-1.5
S1844	23.8	25.5	22.5	26.0	-1.3	0.5
G1845	30.6	34.8	29.3	32.2	-1.3	-2.6
D1846	21.9	23.1	21.9	21.6	0.0	-1.5
R1847	21.8	29.4	21.8	30.0	0.0	0.6
I1848	22.9	27.8	22.4	25.9	-0.5	-1.9
H1849	22.2	23.1	21.9	25.4	-0.3	2.3
C1850	18.4	21.9	18.2	23.7	-0.2	1.8
M1851						
D1852	23.6	34.4	23.7	33.6	0.1	-0.8
I1853						
L1854	25.3	27.4	26.0	26.2	0.7	-1.2
F1855	23.7	25.4	24.5	32.0	0.8	6.6
A1856	23.3	26.2	23.0	23.4	-0.3	-2.8
F1857	23.0	28.5	25.3	27.7	2.3	-0.8
T1858						
K1859	24.3	25.4	22.5	31.3	-1.8	5.9
R1860	21.9	25.2	22.1	24.8	0.2	-0.4
V1861	23.4	29.4	22.8	28.0	-0.6	-1.4
L1862	24.8	23.1	23.7	21.7	-1.1	-1.4
G1863	23.3	22.1	22.4	22.0	-0.9	-0.1
E1864	22.1	23.4	22.2	20.7	0.1	-2.7
S1865	19.9	22.0	19.6	19.3	-0.3	-2.7

Figure S6. Linewidth analysis of CTD-IQ and CTD-IQ + CaM in the absence and presence of Ca^{2+}. Peak shapes that could not be resolved due to overlap were omitted (grey). Data collected on 600 MHz NMR.

$-Ca^{2+}$	0%		10%		20%		50%		100%		200%		400%		800%	
RESID	^{15}N	^{1}H	^{15}N	^{1}H	^{15}N	^{1}H	^{15}N	^{1}H	^{15}N	^{1}H	^{15}N	^{1}H	^{15}N	^{1}H	^{15}N	^{1}H
G-TAG																
S-TAG	115.61	8.09	115.64	8.09	115.61	8.09	115.60	8.09	115.61	8.09	115.58	8.09	115.57	8.09	115.58	8.09
E1773	122.47	8.54	122.47	8.54	122.47	8.54	122.47	8.54	122.47	8.54	122.48	8.54	122.47	8.54	122.50	8.54
N1774	118.89	8.21	118.89	8.21	118.90	8.21	118.90	8.21	118.90	8.21	118.92	8.21	118.96	8.22	118.99	8.22
F1775	120.68	8.02	120.67	8.02	120.67	8.02	120.68	8.02	120.68	8.02	120.70	8.03	120.72	8.03	120.72	8.03
S1776	117.61	8.10	117.63	8.09	117.63	8.09	117.58	8.10	117.59	8.10	117.58	8.10	117.57	8.10	117.57	8.11
V1777	121.51	8.01	121.51	8.01	121.51	8.01	121.51	8.01	121.51	8.01	121.52	8.02	121.52	8.02	121.52	8.02
A1778	127.79	8.30	127.79	8.30	127.79	8.30	127.79	8.30	127.79	8.30	127.79	8.30	127.77	8.30	127.76	8.30
T1779	113.78	8.06	113.78	8.06	113.78	8.06	113.78	8.06	113.78	8.06	113.79	8.06	113.78	8.06	113.77	8.06
E1780	122.99	8.37	122.99	8.37	123.00	8.37	123.00	8.37	123.01	8.37	123.01	8.37	123.01	8.37	123.00	8.37
E1781	121.94	8.36	121.94	8.36	121.94	8.35	121.95	8.36	121.96	8.36	121.99	8.36	122.00	8.37	122.00	8.37
S1782	117.18	8.32	117.18	8.32	117.18	8.32	117.17	8.32	117.17	8.32	117.18	8.32	117.17	8.32	117.14	8.32
T1783	116.47	8.20	116.46	8.20	116.46	8.20	116.46	8.20	116.45	8.20	116.40	8.19	116.35	8.19	116.30	8.18
E1784	124.45	8.34	124.44	8.34	124.44	8.34	124.42	8.34	124.37	8.34	124.23	8.35	124.13	8.35	124.09	8.35
P1785																
L1786	117.79	7.51	117.77	7.50	117.75	7.50	117.68	7.49	117.50	7.46	116.98	7.38				
S1787	119.81	9.50	119.81	9.50	119.81	9.50	119.79	9.50	119.73	9.50	119.59	9.50	119.58	9.50	120.01	9.46
E1788	121.30	8.96	121.29	8.96	121.28	8.96	121.25	8.96	121.19	8.97	120.98	8.97	120.83	8.97	120.77	8.97
D1789	117.10	8.08	117.10	8.08	117.10	8.08	117.09	8.07	117.09	8.07	117.04	8.05	117.01	8.05	117.00	8.05
D1790	120.44	7.68	120.43	7.68	120.43	7.67	120.43	7.67	120.42	7.66	120.35	7.71	120.24	7.71	120.22	7.73
F1791	118.97	7.41	118.96	7.41	118.96	7.41	118.92	7.41	118.82	7.39	OL	OL	OL	OL	OL	OL
D1792	119.51	8.38	119.44	8.39	119.49	8.38	119.50	8.38	119.63	8.38	119.74	8.38	119.34	8.39	119.03	8.38
M1793	120.58	7.80	120.58	7.81	120.55	7.79	120.57	7.82	120.60	7.79	OL	OL	OL	OL	OL	OL
F1794	121.41	7.91	121.36	7.91	121.36	7.91	121.36	7.91	121.40	7.91	120.73	7.93	120.72	7.94	121.11	7.83
Y1795	117.90	8.49	117.92	8.50	117.95	8.49	117.89	8.49	117.91	8.49	117.98	8.50	117.88	8.52	117.89	8.49
E1796	121.48	8.18	121.48	8.18	121.48	8.18	121.48	8.18	121.48	8.18	121.38	8.19				
I1797	119.84	8.15	119.88	8.14	119.88	8.14	119.88	8.14	119.59	8.13	OL	OL	OL	OL	OL	OL
W1798	122.59	9.14	122.60	9.14	122.60	9.14	122.62	9.14	122.66	9.13	122.78	9.11	122.85	9.10	122.87	9.10
E1799	115.60	7.82	115.60	7.82	115.60	7.83	115.59	7.84	115.57	7.86	115.55	7.93	115.54	7.97	115.55	7.97
K1800	116.11	7.35	116.10	7.35	116.14	7.36	116.14	7.36	115.96	7.35	116.27	7.35	116.28	7.36	116.27	7.36
F1801	116.36	7.36	116.32	7.36	116.32	7.36	116.39	7.35	116.31	7.35	116.41	7.36	116.41	7.36	116.43	7.36
D1802	119.81	7.65	119.82	7.65	119.80	7.65	119.81	7.65	119.81	7.65	119.80	7.65	119.79	7.65	119.83	7.64
P1803																
E1804	116.85	8.92	116.85	8.92	116.84	8.92	116.85	8.92	116.84	8.92	116.82	8.93	116.81	8.93	116.80	8.93
A1805	122.41	8.24	122.41	8.24	122.41	8.24	122.40	8.24	122.37	8.24	122.30	8.24	122.24	8.25	122.24	8.25
T1806	114.30	9.75	114.31	9.75	114.30	9.75	114.31	9.75	114.33	9.76	114.37	9.78	114.39	9.79	114.38	9.79
Q1807	113.81	10.47	113.79	10.47	113.78	10.47	113.77	10.47	113.74	10.46	113.63	10.45	113.57	10.45	113.55	10.45
F1808	117.34	8.39	117.33	8.39	117.34	8.39	117.35	8.39	117.36	8.39	117.43	8.40	117.46	8.40	117.48	8.41
I1809	112.63	8.42	112.63	8.43	112.62	8.42	112.62	8.42	112.58	8.42	112.50	8.41	112.47	8.40	112.46	8.41
E1810	122.26	8.31	122.26	8.31	122.25	8.31	122.25	8.31	122.23	8.31	122.17	8.32	122.15	8.32	122.14	8.33
Y1811	123.57	9.05	123.55	9.05	123.57	9.05	123.57	9.05	123.56	9.05	123.54	9.04	123.52	9.04	123.53	9.04
S1812	111.40	8.60	111.39	8.60	111.39	8.59	111.40	8.59	111.39	8.59	111.38	8.59	111.37	8.59	111.38	8.59
V1813	117.13	7.47	117.15	7.47	117.09	7.47	117.09	7.47	117.04	7.48	116.93	7.48	116.81	7.47	116.82	7.47
L1814	124.24	7.64	124.24	7.64	124.24	7.64	124.24	7.64	124.24	7.64	124.23	7.64	124.22	7.64	124.22	7.64
S1815	111.80	8.36	111.80	8.36	111.80	8.36	111.81	8.36	111.82	8.36	111.85	8.36	111.85	8.37	111.86	8.37
D1816	120.36	7.70	120.35	7.69	120.35	7.69	120.34	7.70	120.34	7.70	120.35	7.71	120.35	7.72	120.36	7.72
F1817	122.75	8.08	122.75	8.08	122.76	8.08	122.75	8.08	122.73	8.08	122.68	8.09	122.47	8.10	122.53	8.11
A1818	117.99	8.68	118.00	8.68	117.99	8.68	117.99	8.68	117.99	8.68	118.00	8.69	118.00	8.69	118.00	8.69
D1819	113.30	7.05	113.30	7.05	113.30	7.05	113.30	7.05	113.30	7.05	113.29	7.05	113.28	7.05	113.29	7.05
A1820	120.20	7.34	120.21	7.34	120.21	7.34	120.21	7.34	120.21	7.34	120.21	7.34	120.21	7.34	120.22	7.34
L1821	115.96	6.25	115.97	6.25	115.97	6.25	115.96	6.25	115.93	6.24	115.87	6.24	115.82	6.24	115.80	6.23
S1822	113.63	8.00	113.62	8.00	113.63	8.00	113.61	8.00	113.59	8.00	113.61	8.02	113.51	7.98	113.56	7.97
E1823	124.99	9.07	124.99	9.07	125.00	9.07	125.00	9.07	125.01	9.07	125.03	9.07	125.07	9.08	125.10	9.09
P1824																
L1825	122.32	7.54	122.32	7.54	122.33	7.54	122.33	7.54	122.30	7.53	122.28	7.53	122.28	7.53	122.31	7.54
R1826	115.48	6.63	115.48	6.63	115.48	6.63	115.48	6.63	115.46	6.63	115.42	6.64	115.38	6.64	115.35	6.64
I1827	125.84	7.07	125.84	7.07	125.83	7.07	125.83	7.07	125.80	7.06	125.76	7.05	125.72	7.03	125.71	7.03
A1828	129.20	8.41	129.22	8.40	129.20	8.41	129.22	8.40	129.21	8.41	129.22	8.40	129.24	8.40	129.24	8.40
K1829	119.82	8.33	119.83	8.33	119.82	8.33	119.82	8.33	119.82	8.34	119.82	8.34	119.83	8.35	119.82	8.35
P1830																
N1831	120.94	8.07	120.92	8.07	120.92	8.07	120.92	8.07	120.97	8.07	120.97	8.07	120.88	8.07	120.85	8.07
Q1832	120.82	8.16	120.81	8.16	120.82	8.16	120.82	8.16	120.84	8.16	120.86	8.16	120.88	8.15	120.86	8.16
I1833	118.25	8.22	118.25	8.21	118.25	8.21	118.24	8.21	118.25	8.21	118.23	8.21	118.22	8.21	118.23	8.22
S1834	115.41	7.45	115.42	7.45	115.41	7.45	115.41	7.45	115.42	7.45	115.40	7.46	115.40	7.45	115.45	7.46
L1835	119.83	7.64	119.82	7.64	119.83	7.64	119.83	7.64	119.82	7.64	119.81	7.64	119.81	7.64	119.83	7.64
I1836	121.72	8.33	121.77	8.32	121.70	8.33	121.77	8.32	121.76	8.34	121.68	8.32	121.72	8.33	121.75	8.33
N1837	117.44	7.73	117.45	7.73	117.45	7.73	117.45	7.73	117.45	7.73	117.44	7.72	117.44	7.72	117.43	7.72
M1838	118.49	7.38	118.49	7.38	118.48	7.38	118.48	7.38	118.49	7.38	118.51	7.37	118.48	7.37	118.49	7.37
D1839	119.55	7.92	119.55	7.92	119.56	7.92	119.56	7.92	119.58	7.92	119.63	7.93	119.70	7.93	119.91	7.94
L1840	121.80	8.73	121.80	8.73	121.80	8.73	121.81	8.73	121.82	8.73	121.88	8.72	121.93	8.72	121.94	8.70
P1841																
M1842	121.56	8.23	121.56	8.23	121.55	8.23	121.53	8.22	121.52	8.24	121.49	8.24	121.47	8.24	121.48	8.24
V1843	118.61	8.93	118.60	8.93	118.60	8.93	118.61	8.93	118.59	8.93	118.56	8.92	118.54	8.92	118.53	8.92
S1844	114.98	8.14	114.98	8.14	114.99	8.14	114.98	8.14	114.99	8.14	114.98	8.14	114.99	8.14	115.01	8.14
G1845	111.76	8.66	111.78	8.66	111.77	8.66	111.80	8.66	111.75	8.65	111.76	8.65	111.76	8.66	111.74	8.66
D1846	119.07	8.29	119.07	8.30	119.08	8.29	119.07	8.29	119.08	8.29	119.09	8.29	119.11	8.29	119.10	8.29
R1847	116.94	7.24	116.95	7.24	116.94	7.24	116.94	7.24	116.95	7.24	117.01	7.25	116.99	7.24	116.99	7.25
I1848	120.07	9.20	120.07	9.20	120.07	9.20	120.07	9.20	120.06	9.20	120.04	9.20	120.03	9.20	120.02	9.21
H1849	128.80	9.77	128.80	9.77	128.80	9.77	128.80	9.77	128.79	9.77	128.76	9.78	128.72	9.78	128.74	9.78
C1850	125.34	7.91	125.28	7.88	125.31	7.89	125.35	7.88	125.31	7.88	125.36	7.87	125.33	7.88	125.40	7.92
M1851																
D1852	118.93	7.23	118.92	7.23	118.90	7.23	118.86	7.22	118.76	7.20	118.43	7.14	118.40	7.18	118.42	7.19
I1853	122.11	7.42	122.26	7.44	122.06	7.42	122.02	7.42	122.05	7.42	OL	OL	OL	OL	OL	OL
L1854	120.42	8.62	120.42	8.62	120.41	8.62	120.40	8.62	120.36	8.60	120.22	8.57	120.16	8.55	120.18	8.55
F1855	119.65	8.09	119.65	8.10	119.65	8.10	119.63	8.11	119.62	8.08	119.56	8.13	119.60	8.16	119.46	8.09
A1856	121.32	7.85	121.32	7.85	121.31	7.85	121.29	7.85	121.25	7.84	121.14	7.83	121.08	7.82	121.03	7.87
F1857	119.39	9.51	119.39	9.51	119.39	9.51	119.39	9.51	119.41	9.51	119.43	9.50	119.42	9.50	119.48	9.51
T1858	115.90	8.23	115.90	8.23	115.93	8.23	116.14	8.24	116.33	8.24	116.47	8.25	116.47	8.24	OL	OL
K1859	122.65	7.92	122.65	7.92	122.64	7.92	122.62	7.94	122.58	7.96	122.30	7.94	122.37	8.11	122.37	8.12
R1860	117.51	7.28	117.51	7.28	117.55	7.29	117.49	7.28	117.45	7.28	117.34	7.27	117.26	7.28	117.26	7.28
V1861	117.54	7.01	117.54	7.01	117.54	7.01	117.56	7.01	117.60	7.01	117.73	7.01	117.79	7.01	117.77	7.00
L1862	118.76	7.90	118.75	7.90	118.74	7.90	118.70	7.89	118.59	7.89	118.20	7.86	118.02	7.86	119.99	7.92
G1863	106.99	7.87	106.99	7.87	106.99	7.87	107.00	7.87	107.03	7.86	107.12	7.85	107.12	7.84	107.04	7.84
E1864	120.06	7.95	120.06	7.95	120.06	7.95	120.06	7.95	120.06	7.95	120.03	7.94	120.01	7.93	119.99	7.92
S1865	122.30	7.88	122.31	7.88	122.31	7.88	122.31	7.89	122.33	7.89	122.34	7.89	122.33	7.89	122.31	7.89

Figure S7. (A) Table of NMR resonance frequencies of CTD in the presence of CaM while adding aliquots of IQ motif peptide in the absence of Ca^{2+} .

+Ca^{2+}	0%		10%		20%		50%		100%		200%		400%		800%	
RESID	^{15}N	^{1}H	^{15}N	^{1}H	^{15}N	^{1}H	^{15}N	^{1}H	^{15}N	^{1}H	^{15}N	^{1}H	^{15}N	^{1}H	^{15}N	^{1}H
G-TAG																
S-TAG	115.59	8.09	115.58	8.08	115.57	8.08	115.58	8.08	115.59	8.08	115.59	8.09	115.58	8.08	115.59	8.09
E1773	122.48	8.53	122.47	8.54	122.47	8.53	122.47	8.54	122.48	8.54	122.48	8.54	122.48	8.54	122.48	8.54
N1774	118.91	8.21	118.91	8.20	118.91	8.20	118.90	8.21	118.89	8.21	118.93	8.21	118.95	8.21	118.95	8.21
F1775	120.66	8.01	120.65	8.01	120.65	8.01	120.66	8.01	120.66	8.01	120.67	8.02	120.69	8.03	120.69	8.02
S1776	117.54	8.09	117.53	8.09	117.53	8.09	117.54	8.09	117.53	8.09	117.54	8.09	117.54	8.09	117.53	8.10
V1777	121.47	8.00	121.47	8.00	121.47	8.00	121.47	8.00	121.47	8.00	121.48	8.00	121.49	8.01	121.50	8.01
A1778	127.71	8.29	127.71	8.29	127.71	8.29	127.71	8.29	127.72	8.29	127.73	8.29	127.72	8.29	127.72	8.29
T1779	113.70	8.04	113.69	8.04	113.69	8.04	113.69	8.04	113.70	8.04	113.71	8.04	113.72	8.05	113.72	8.05
E1780	123.02	8.36	123.02	8.36	123.02	8.36	123.02	8.36	123.02	8.36	123.03	8.37	123.02	8.37	123.01	8.37
E1781	121.97	8.36	121.97	8.36	121.97	8.36	121.97	8.36	121.97	8.36	121.99	8.36	122.00	8.37	122.00	8.37
S1782	117.06	8.31	117.04	8.30	117.04	8.30	117.05	8.31	117.06	8.31	117.06	8.31	117.06	8.31	117.05	8.31
T1783	116.32	8.17	116.32	8.17	116.32	8.17	116.32	8.17	116.32	8.17	116.29	8.17	116.25	8.17	116.22	8.17
E1784	124.40	8.29	124.40	8.29	124.40	8.29	124.40	8.29	124.39	8.29	124.30	8.30	124.18	8.31	124.11	8.31
P1785																
L1786	118.26	7.60	118.26	7.60	118.26	7.60	118.26	7.59	118.22	7.59	117.92	7.54	117.57	7.48		
S1787	119.89	9.47	119.89	9.47	119.88	9.47	119.89	9.47	119.87	9.47	119.76	9.46	119.56	9.48	119.53	9.50
E1788	121.21	8.95	121.21	8.95	121.21	8.95	121.21	8.95	121.19	8.96	121.07	8.96	120.92	8.97	120.83	8.97
D1789	116.98	8.13	116.98	8.13	116.98	8.13	116.98	8.13	116.97	8.13	116.91	8.12	116.84	8.10	116.81	8.09
D1790	120.52	7.68	120.59	7.69	120.62	7.68	120.60	7.69	120.58	7.68	120.50	7.67	120.39	7.65	120.26	7.73
F1791	118.94	7.35	118.94	7.35	118.94	7.35	118.93	7.35	118.91	7.35	118.72	7.33	118.53	7.31	118.46	7.31
D1792	119.39	8.37	119.37	8.37	119.32	8.37	119.35	8.38	119.37	8.37	119.39	8.37	119.84	8.34	119.62	8.37
M1793	120.31	7.78	120.26	7.78	120.26	7.78	120.30	7.78	120.27	7.79	120.71	7.80	120.34	7.78	120.62	7.83
F1794	121.74	7.91	121.72	7.91	121.77	7.91	121.80	7.91	121.72	7.91	121.73	7.91	121.60	7.92	121.45	7.94
Y1795	117.79	8.45	117.78	8.45	117.79	8.46	117.77	8.46	117.77	8.45	117.87	8.45	117.69	8.51	OL	OL
E1796	121.38	8.19	121.36	8.19	121.42	8.17	121.39	8.17	121.37	8.17	OL	OL	OL	OL	OL	OL
I1797	119.80	8.16	119.79	8.15	119.78	8.15	119.78	8.15	119.74	8.15	OL	OL	OL	OL	OL	OL
W1798	122.62	9.14	122.62	9.14	122.63	9.14	122.63	9.13	122.64	9.13	122.72	9.12	122.83	9.10	122.86	9.10
E1799	115.53	7.81	115.52	7.81	115.52	7.81	115.52	7.81	115.52	7.82	115.50	7.87	115.27	7.79	115.52	7.95
K1800	116.07	7.34	115.96	7.34	116.22	7.34	116.15	7.34	116.12	7.34	116.18	7.34	116.01	7.34	116.05	7.35
F1801	116.25	7.34	116.25	7.34	116.25	7.34	116.26	7.34	116.26	7.34	116.30	7.35	116.37	7.35	116.37	7.35
D1802	119.76	7.67	119.76	7.67	119.75	7.67	119.75	7.67	119.74	7.67	119.72	7.67	119.72	7.67	119.69	7.66
P1803																
E1804	116.85	8.92	116.85	8.92	116.85	8.92	116.85	8.92	116.84	8.92	116.83	8.92	116.82	8.92	116.80	8.92
A1805	122.36	8.24	122.36	8.23	122.36	8.24	122.36	8.24	122.35	8.24	122.30	8.24	122.24	8.24	122.19	8.25
T1806	114.31	9.74	114.30	9.74	114.31	9.74	114.30	9.74	114.31	9.74	114.33	9.76	114.37	9.78	114.37	9.78
Q1807	113.70	10.46	113.69	10.45	113.69	10.45	113.69	10.45	113.67	10.45	113.60	10.44	113.53	10.44	113.49	10.44
F1808	117.34	8.39	117.34	8.38	117.35	8.39	117.34	8.38	117.34	8.38	117.39	8.39	117.45	8.40	117.46	8.40
I1809	112.58	8.42	112.57	8.42	112.59	8.42	112.59	8.42	112.58	8.42	112.53	8.41	112.46	8.40	112.43	8.39
E1810	122.18	8.30	122.18	8.30	122.18	8.30	122.19	8.30	122.19	8.30	122.16	8.31	122.10	8.31	122.15	8.30
Y1811	123.56	9.04	123.56	9.04	123.56	9.04	123.56	9.04	123.55	9.04	123.54	9.04	123.52	9.04	123.52	9.04
S1812	111.36	8.59	111.36	8.59	111.36	8.59	111.37	8.59	111.37	8.59	111.36	8.59	111.35	8.58	111.35	8.58
V1813	117.04	7.47	117.03	7.48	117.01	7.48	117.02	7.47	116.99	7.47	116.94	7.47	116.84	7.47	116.85	7.47
L1814	124.23	7.63	124.23	7.63	124.23	7.63	124.23	7.63	124.23	7.63	124.21	7.63	124.22	7.63	124.20	7.63
S1815	111.82	8.37	111.81	8.37	111.82	8.37	111.82	8.37	111.82	8.37	111.84	8.37	111.87	8.37	111.88	8.37
D1816	120.41	7.69	120.37	7.70	120.38	7.70	120.36	7.70	120.36	7.70	120.34	7.71	120.34	7.72	120.39	7.72
F1817	122.72	8.08	122.71	8.08	122.71	8.08	122.71	8.08	122.71	8.08	122.67	8.09	122.55	8.10	122.57	8.11
A1818	117.96	8.68	117.96	8.67	117.96	8.67	117.95	8.67	117.96	8.67	117.96	8.68	117.96	8.68	117.96	8.69
D1819	113.29	7.05	113.28	7.05	113.28	7.05	113.28	7.05	113.28	7.05	113.28	7.05	113.26	7.05	113.27	7.05
A1820	120.23	7.33	120.22	7.33	120.23	7.33	120.23	7.33	120.22	7.33	120.23	7.33	120.23	7.33	120.23	7.33
L1821	115.78	6.23	115.78	6.22	115.77	6.22	115.77	6.22	115.77	6.22	115.73	6.22	115.71	6.22	115.72	6.22
S1822	113.42	7.95	113.42	7.95	113.41	7.94	113.42	7.95	113.41	7.95	113.42	7.95	113.44	7.97	113.43	7.98
E1823	124.87	9.06	124.88	9.06	124.87	9.06	124.88	9.06	124.88	9.06	124.90	9.07	124.94	9.07	125.00	9.08
P1824																
L1825	122.13	7.49	122.13	7.49	122.12	7.49	122.12	7.49	122.10	7.49	121.89	7.47	121.68	7.45	121.59	7.43
R1826	115.18	6.67	115.19	6.67	115.19	6.67	115.19	6.67	115.19	6.67	115.16	6.68	115.12	6.68	115.16	6.67
I1827	126.18	7.05	126.18	7.05	126.18	7.05	126.18	7.04	126.17	7.05	126.15	7.05	126.10	7.04	126.05	7.04
A1828	129.25	8.40	129.26	8.40	129.26	8.40	129.26	8.40	129.26	8.40	129.27	8.40	129.27	8.40	129.27	8.40
K1829	119.81	8.33	119.81	8.33	119.81	8.33	119.81	8.33	119.81	8.33	119.83	8.34	119.84	8.34	119.82	8.35
P1830																
N1831	120.95	8.07	120.93	8.07	120.95	8.07	120.94	8.08	120.96	8.08	120.95	8.07	120.96	8.07	120.92	8.07
Q1832	120.85	8.16	120.85	8.16	120.85	8.16	120.85	8.15	120.85	8.16	120.86	8.16	120.87	8.16	120.87	8.16
I1833	118.25	8.21	118.24	8.21	118.24	8.21	118.24	8.21	118.24	8.21	118.24	8.21	118.23	8.21	118.25	8.21
S1834	115.44	7.47	115.43	7.47	115.43	7.47	115.43	7.47	115.45	7.47	115.45	7.47	115.40	7.47	115.42	7.48
L1835	119.81	7.64	119.80	7.64	119.81	7.64	119.81	7.64	119.81	7.64	119.80	7.64	119.79	7.64	119.80	7.64
I1836	121.80	8.35	121.77	8.35	121.76	8.35	121.77	8.35	121.77	8.35	121.76	8.35	121.81	8.34	121.76	8.35
N1837	117.44	7.74	117.43	7.74	117.44	7.74	117.44	7.74	117.44	7.74	117.44	7.74	117.44	7.73	117.43	7.72
M1838	118.50	7.38	118.49	7.38	118.49	7.38	118.49	7.38	118.49	7.38	118.50	7.37	118.49	7.37	118.49	7.37
D1839	119.48	7.91	119.49	7.91	119.48	7.91	119.50	7.91	119.49	7.91	119.52	7.92	119.57	7.93	119.59	7.94
L1840	121.75	8.74	121.74	8.73	121.74	8.74	121.74	8.73	121.75	8.73	121.78	8.73	121.85	8.72	121.85	8.72
P1841																
M1842	121.50	8.22	121.50	8.22	121.50	8.22	121.50	8.22	121.50	8.22	121.46	8.22	121.44	8.22	121.65	8.22
V1843	118.60	8.92	118.59	8.92	118.60	8.92	118.60	8.92	118.59	8.92	118.57	8.92	118.54	8.92	118.52	8.92
S1844	114.98	8.14	114.98	8.14	114.98	8.14	114.98	8.14	114.99	8.14	114.99	8.14	115.00	8.14	115.02	8.14
G1845	111.72	8.66	111.73	8.66	111.71	8.66	111.72	8.65	111.74	8.65	111.74	8.65	111.75	8.66	111.73	8.65
D1846	119.09	8.29	119.08	8.29	119.08	8.29	119.08	8.29	119.08	8.29	119.09	8.28	119.10	8.28	119.12	8.29
R1847	116.94	7.24	116.94	7.24	116.93	7.24	116.93	7.24	116.93	7.24	116.95	7.24	116.99	7.25	117.01	7.25
I1848	120.07	9.19	120.07	9.19	120.07	9.19	120.08	9.19	120.07	9.19	120.07	9.19	120.05	9.20	120.03	9.20
H1849	128.83	9.77	128.82	9.78	128.82	9.78	128.82	9.78	128.82	9.77	128.81	9.78	128.78	9.78	128.77	9.78
C1850	125.26	7.88	125.27	7.87	125.29	7.86	125.29	7.89	125.26	7.86	125.21	7.86	125.27	7.86		
M1851																
D1852	118.90	7.21	118.89	7.21	118.90	7.21	118.88	7.21	118.84	7.20	118.61	7.16	118.51	7.23	118.47	7.25
I1853	121.99	7.40	121.99	7.41	122.02	7.42	122.03	7.41	122.03	7.40	121.75	7.42				
L1854	120.34	8.61	120.39	8.61	120.40	8.61	120.39	8.61	120.38	8.61	120.24	8.57	120.18	8.54	120.15	8.53
F1855	119.66	8.11	119.66	8.11	119.66	8.11	119.66	8.11	119.65	8.12	119.57	8.10	119.48	8.14	119.37	8.04
A1856	121.35	7.86	121.38	7.85	121.34	7.85	121.34	7.85	121.32	7.85	121.23	7.84	121.12	7.82	121.10	7.83
F1857	119.47	9.52	119.47	9.51	119.47	9.52	119.47	9.51	119.46	9.51	119.45	9.50	119.47	9.50	119.38	9.50
T1858	116.18	8.23	116.15	8.23	116.19	8.23	116.20	8.21	116.11	8.21	116.43	8.22	115.77	8.25	OL	OL
K1859	122.62	7.94	122.61	7.94	122.61	7.94	122.61	7.94	122.61	7.95	122.52	8.03	122.38	8.11	122.46	8.12
R1860	117.54	7.30	117.53	7.30	117.54	7.30	117.54	7.30	117.53	7.30	117.44	7.29	117.37	7.29	117.31	7.29
V1861	117.55	7.00	117.54	7.00	117.55	7.00	117.54	7.00	117.56	7.00	117.66	7.00	117.78	7.01	117.79	7.00
L1862	118.74	7.92	118.74	7.92	118.74	7.92	118.74	7.92	118.70	7.91	118.45	7.90	118.13	7.88	118.00	7.87
G1863	106.90	7.87	106.90	7.87	106.90	7.87	106.90	7.87	106.91	7.87	106.95	7.85	107.04	7.84	107.03	7.84
E1864	120.11	7.92	120.11	7.92	120.10	7.92	120.10	7.92	120.10	7.92	120.10	7.91	120.07	7.91	120.04	7.90
S1865	122.18	7.86	122.17	7.85	122.17	7.86	122.18	7.86	122.18	7.86	122.20	7.86	122.23	7.87	122.24	7.87

(B) Table of NMR resonance frequencies of CTD in the presence of CaM while adding aliquots of IQ motif peptide in the presence Ca^{2+} .

$-Ca^{2+}$ RESID	10% Δ1vs2	20% Δ1vs3	50% Δ1vs4	100% Δ1vs5	200% Δ1vs6	400% Δ1vs7	800% Δ1vs8
G-TAG							
S-TAG	0.012	0.003	0.003	0.001	0.009	0.011	0.009
E1773	0.001	0.001	0.002	0.001	0.002	0.001	0.009
N1774	0.001	0.002	0.003	0.003	0.009	0.023	0.032
F1775	0.001	0.001	0.001	0.003	0.010	0.016	0.017
S1776	0.007	0.007	0.007	0.006	0.009	0.013	0.014
V1777	0.001	0.000	0.001	0.003	0.005	0.005	0.005
A1778	0.000	0.000	0.001	0.002	0.002	0.005	0.009
T1779	0.000	0.001	0.002	0.002	0.004	0.001	0.003
E1780	0.000	0.001	0.002	0.004	0.006	0.006	0.004
E1781	0.001	0.002	0.003	0.008	0.018	0.021	0.021
S1782	0.000	0.000	0.002	0.001	0.002	0.003	0.011
T1783	0.002	0.002	0.003	0.006	0.021	0.038	0.055
E1784	0.001	0.003	0.009	0.023	0.069	0.099	0.114
P1785							
L1786	0.006	0.013	0.035	0.096	0.271		
S1787	0.002	0.001	0.006	0.027	0.071	0.072	0.066
E1788	0.003	0.005	0.013	0.035	0.099	0.147	0.167
D1789	0.001	0.001	0.002	0.007	0.025	0.036	0.037
D1790	0.001	0.001	0.005	0.011	0.039	0.068	0.078
F1791	0.004	0.003	0.016	0.049	OL	OL	OL
D1792	0.021	0.007	0.001	0.039	0.076	0.052	0.152
M1793	0.002	0.014	0.009	0.011	OL	OL	OL
F1794	0.015	0.015	0.015	0.003	0.217	0.220	0.112
Y1795	0.007	0.015	0.002	0.004	0.025	0.018	0.005
E1796	0.002	0.000	0.003	0.002	0.030		
I1797	0.015	0.015	0.015	0.078	OL	OL	OL
W1798	0.001	0.003	0.010	0.022	0.062	0.085	0.092
E1799	0.002	0.004	0.012	0.029	0.079	0.106	0.108
K1800	0.002	0.011	0.011	0.048	0.051	0.055	0.051
F1801	0.012	0.012	0.010	0.016	0.016	0.016	0.021
D1802	0.006	0.004	0.003	0.003	0.004	0.007	0.008
P1803							
E1804	0.000	0.001	0.001	0.003	0.010	0.013	0.016
A1805	0.000	0.002	0.005	0.013	0.035	0.056	0.056
T1806	0.002	0.001	0.004	0.011	0.031	0.040	0.038
Q1807	0.004	0.007	0.011	0.022	0.057	0.078	0.081
F1808	0.002	0.001	0.003	0.007	0.029	0.038	0.047
I1809	0.001	0.003	0.005	0.017	0.044	0.055	0.056
E1810	0.001	0.001	0.003	0.008	0.028	0.036	0.038
Y1811	0.009	0.001	0.002	0.003	0.011	0.018	0.013
S1812	0.001	0.001	0.001	0.002	0.006	0.009	0.008
V1813	0.007	0.015	0.015	0.030	0.065	0.101	0.100
L1814	0.001	0.000	0.001	0.001	0.004	0.006	0.007
S1815	0.001	0.001	0.002	0.006	0.016	0.018	0.020
D1816	0.002	0.002	0.006	0.008	0.014	0.017	0.016
F1817	0.001	0.003	0.002	0.008	0.024	0.091	0.073
A1818	0.002	0.001	0.001	0.001	0.005	0.004	0.007
D1819	0.002	0.001	0.002	0.001	0.005	0.008	0.005
A1820	0.001	0.001	0.001	0.001	0.002	0.002	0.004
L1821	0.003	0.002	0.000	0.010	0.029	0.044	0.050
S1822	0.002	0.001	0.005	0.011	0.014	0.037	0.027
E1823	0.000	0.001	0.001	0.005	0.012	0.026	0.038
P1824							
L1825	0.001	0.003	0.003	0.007	0.015	0.015	0.002
R1826	0.000	0.000	0.001	0.007	0.020	0.035	0.042
I1827	0.001	0.003	0.004	0.012	0.029	0.044	0.049
A1828	0.006	0.000	0.006	0.002	0.007	0.011	0.012
K1829	0.001	0.001	0.001	0.003	0.007	0.012	0.009
P1830							
N1831	0.007	0.007	0.007	0.009	0.009	0.019	0.029
Q1832	0.003	0.001	0.001	0.007	0.013	0.017	0.012
I1833	0.001	0.001	0.002	0.002	0.005	0.008	0.005
S1834	0.004	0.001	0.001	0.003	0.005	0.003	0.012
L1835	0.002	0.001	0.002	0.003	0.006	0.007	0.004
I1836	0.016	0.006	0.016	0.017	0.012	0.005	0.013
N1837	0.004	0.004	0.004	0.003	0.006	0.009	0.010
M1838	0.000	0.001	0.001	0.002	0.007	0.004	0.002
D1839	0.001	0.002	0.003	0.009	0.025	0.047	0.114
L1840	0.002	0.001	0.002	0.007	0.027	0.041	0.048
P1841							
M1842	0.001	0.000	0.016	0.011	0.021	0.027	0.025
V1843	0.001	0.002	0.001	0.005	0.016	0.021	0.025
S1844	0.001	0.001	0.001	0.002	0.001	0.002	0.008
G1845	0.004	0.001	0.010	0.005	0.004	0.002	0.009
D1846	0.001	0.001	0.000	0.002	0.007	0.011	0.009
R1847	0.002	0.002	0.001	0.002	0.021	0.017	0.017
I1848	0.001	0.001	0.001	0.001	0.010	0.013	0.017
H1849	0.001	0.002	0.003	0.005	0.014	0.028	0.021
C1850	0.027	0.013	0.019	0.024	0.025	0.019	0.022
M1851							
D1852	0.003	0.010	0.025	0.060	0.170	0.171	0.164
I1853	0.049	0.018	0.029	0.020	OL	OL	OL
L1854	0.001	0.004	0.010	0.025	0.074	0.098	0.093
F1855	0.005	0.006	0.011	0.015	0.036	0.053	0.062
A1856	0.001	0.003	0.009	0.023	0.059	0.078	0.092
F1857	0.001	0.000	0.002	0.009	0.017	0.012	0.030
T1858	0.003	0.009	0.078	0.138	0.181	0.182	OL
K1859	0.004	0.006	0.018	0.041	0.112	0.162	0.170
R1860	0.002	0.014	0.008	0.022	0.055	0.080	0.080
V1861	0.000	0.002	0.008	0.019	0.061	0.079	0.073
L1862	0.004	0.007	0.022	0.057	0.179	0.238	0.389
G1863	0.001	0.001	0.004	0.014	0.043	0.049	0.029
E1864	0.000	0.001	0.001	0.003	0.013	0.022	0.030
S1865	0.005	0.005	0.002	0.007	0.014	0.010	0.007

$+Ca^{2+}$ RESID	10% Δ11vs12	20% Δ11vs13	50% Δ11vs14	100% Δ11vs15	200% Δ11vs16	400% Δ11vs17	800% Δ11vs18
G-TAG							
S-TAG	0.004	0.006	0.004	0.002	0.002	0.005	0.005
E1773	0.002	0.001	0.001	0.002	0.001	0.002	0.004
N1774	0.002	0.002	0.004	0.006	0.008	0.015	0.012
F1775	0.002	0.002	0.001	0.001	0.004	0.014	0.011
S1776	0.004	0.002	0.002	0.002	0.003	0.003	0.008
V1777	0.001	0.001	0.001	0.001	0.004	0.009	0.011
A1778	0.002	0.001	0.000	0.001	0.004	0.004	0.006
T1779	0.001	0.003	0.001	0.000	0.003	0.007	0.008
E1780	0.002	0.002	0.001	0.000	0.001	0.003	0.006
E1781	0.001	0.001	0.001	0.003	0.006	0.010	0.010
S1782	0.004	0.004	0.003	0.003	0.003	0.001	0.002
T1783	0.001	0.001	0.001	0.000	0.009	0.021	0.030
E1784	0.001	0.002	0.001	0.005	0.034	0.073	0.095
P1785							
L1786	0.001	0.001	0.003	0.016	0.115	0.234	
S1787	0.002	0.002	0.002	0.004	0.040	0.103	0.114
E1788	0.002	0.000	0.000	0.006	0.043	0.093	0.121
D1789	0.001	0.002	0.003	0.004	0.023	0.051	0.061
D1790	0.023	0.031	0.025	0.021	0.013	0.049	0.088
F1791	0.001	0.002	0.001	0.009	0.070	0.132	0.155
D1792	0.006	0.023	0.013	0.008	0.002	0.145	0.074
M1793	0.014	0.015	0.002	0.011	0.129	0.011	0.105
F1794	0.007	0.010	0.018	0.007	0.003	0.047	0.094
Y1795	0.004	0.005	0.007	0.006	0.027	0.054	OL
E1796	0.006	0.018	0.011	0.013	OL	OL	OL
I1797	0.005	0.006	0.007	0.021	OL	OL	OL
W1798	0.000	0.002	0.002	0.005	0.032	0.070	0.080
E1799	0.003	0.004	0.003	0.007	0.045	0.082	0.100
K1800	0.034	0.047	0.025	0.017	0.035	0.018	0.007
F1801	0.001	0.001	0.002	0.004	0.016	0.038	0.039
D1802	0.001	0.002	0.003	0.005	0.010	0.013	0.023
P1803							
E1804	0.002	0.000	0.000	0.003	0.008	0.011	0.018
A1805	0.002	0.002	0.001	0.004	0.021	0.041	0.055
T1806	0.002	0.001	0.001	0.001	0.013	0.031	0.032
Q1807	0.004	0.004	0.004	0.010	0.033	0.053	0.066
F1808	0.001	0.002	0.002	0.001	0.016	0.035	0.037
I1809	0.003	0.003	0.003	0.001	0.018	0.037	0.049
E1810	0.000	0.001	0.003	0.002	0.007	0.027	0.011
Y1811	0.002	0.001	0.002	0.004	0.007	0.013	0.015
S1812	0.001	0.002	0.002	0.002	0.003	0.006	0.006
V1813	0.004	0.008	0.006	0.016	0.029	0.062	0.060
L1814	0.002	0.001	0.001	0.001	0.007	0.004	0.009
S1815	0.002	0.001	0.001	0.001	0.005	0.014	0.018
D1816	0.012	0.011	0.016	0.015	0.024	0.028	0.019
F1817	0.003	0.002	0.003	0.002	0.014	0.055	0.051
A1818	0.002	0.002	0.003	0.001	0.000	0.004	0.008
D1819	0.001	0.001	0.001	0.002	0.003	0.007	0.006
A1820	0.002	0.000	0.001	0.003	0.002	0.002	0.001
L1821	0.001	0.002	0.001	0.002	0.014	0.020	0.018
S1822	0.001	0.004	0.002	0.003	0.006	0.018	0.023
E1823	0.001	0.000	0.001	0.003	0.010	0.024	0.042
P1824							
L1825	0.001	0.001	0.001	0.010	0.077	0.145	0.176
R1826	0.002	0.002	0.003	0.003	0.007	0.020	0.007
I1827	0.001	0.003	0.001	0.003	0.012	0.028	0.042
A1828	0.001	0.001	0.001	0.001	0.004	0.007	0.006
K1829	0.000	0.001	0.000	0.001	0.007	0.013	0.011
P1830							
N1831	0.008	0.004	0.003	0.004	0.001	0.002	0.009
Q1832	0.001	0.002	0.001	0.001	0.003	0.007	0.006
I1833	0.002	0.002	0.002	0.002	0.003	0.003	0.001
S1834	0.003	0.003	0.003	0.004	0.006	0.010	0.007
L1835	0.001	0.001	0.001	0.001	0.002	0.005	0.002
I1836	0.012	0.013	0.011	0.011	0.013	0.012	0.015
N1837	0.001	0.000	0.001	0.001	0.004	0.009	0.014
M1838	0.001	0.003	0.001	0.001	0.002	0.002	0.004
D1839	0.002	0.001	0.004	0.003	0.013	0.031	0.038
L1840	0.006	0.006	0.007	0.004	0.012	0.034	0.034
P1841							
M1842	0.002	0.001	0.001	0.000	0.013	0.021	0.048
V1843	0.002	0.002	0.001	0.003	0.012	0.019	0.025
S1844	0.003	0.002	0.001	0.002	0.002	0.004	0.011
G1845	0.003	0.001	0.007	0.009	0.008	0.011	0.005
D1846	0.001	0.001	0.002	0.001	0.001	0.005	0.009
R1847	0.001	0.001	0.001	0.001	0.004	0.019	0.024
I1848	0.002	0.001	0.002	0.001	0.002	0.007	0.013
H1849	0.002	0.003	0.004	0.004	0.006	0.017	0.018
C1850	0.010	0.015	0.010	0.014	0.021	0.014	
M1851							
D1852	0.005	0.003	0.005	0.017	0.096	0.125	0.140
I1853	0.004	0.014	0.012	0.011	0.079		
L1854	0.017	0.019	0.018	0.014	0.045	0.071	0.084
F1855	0.001	0.002	0.001	0.011	0.028	0.060	0.104
A1856	0.011	0.002	0.002	0.009	0.039	0.075	0.080
F1857	0.003	0.003	0.001	0.006	0.012	0.014	0.030
T1858	0.007	0.005	0.013	0.025	0.080	0.128	OL
K1859	0.004	0.004	0.003	0.008	0.068	0.139	0.137
R1860	0.001	0.001	0.001	0.003	0.030	0.054	0.071
V1861	0.003	0.001	0.003	0.003	0.035	0.074	0.075
L1862	0.001	0.001	0.001	0.013	0.095	0.195	0.238
G1863	0.001	0.001	0.001	0.004	0.021	0.051	0.048
E1864	0.001	0.001	0.001	0.001	0.003	0.013	0.024
S1865	0.001	0.001	0.001	0.001	0.009	0.020	0.025

(C) Delta values for CTD amino acids for each addition of IQ motif peptide in the absence (left) and presence of Ca^{2+} (right). Values were calculated using $\Delta = [0.5 \times ({}^{1}H_a - {}^{1}H_b)^2 + (0.2 \times ({}^{15}N_a - {}^{15}N_b)^2)]^{0.5}$ Williamson et al 2013. Color code: **< 0.05 ppm**, **>0.05 < 0.09**, **> 0.09 < 0.02**, **> 0.20 ppm** **Peak disappearance**, Unable to track assignment due to cross-peak overlap **(OL)**.

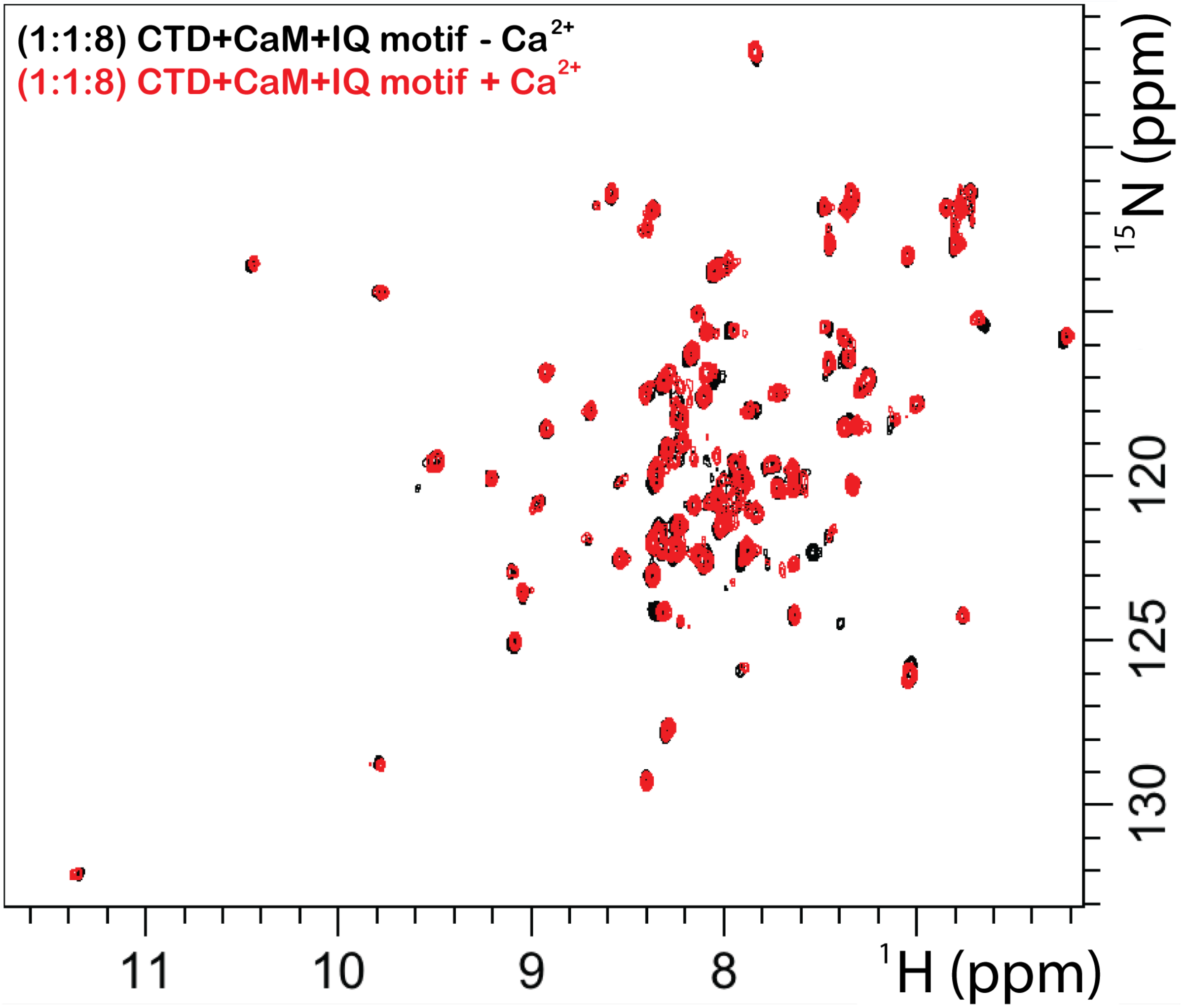

Figure S8. Overlay of ^{15}N CTD- CaM spectra containing 800% IQ motif peptide in the absence **(black)** and presence of Ca^{2+} (**red**). The high degree of similarity between the spectra was consistent with the CTD-IQ motif forming the same complex irrespective of the presence of Ca^{2+} or CaM.

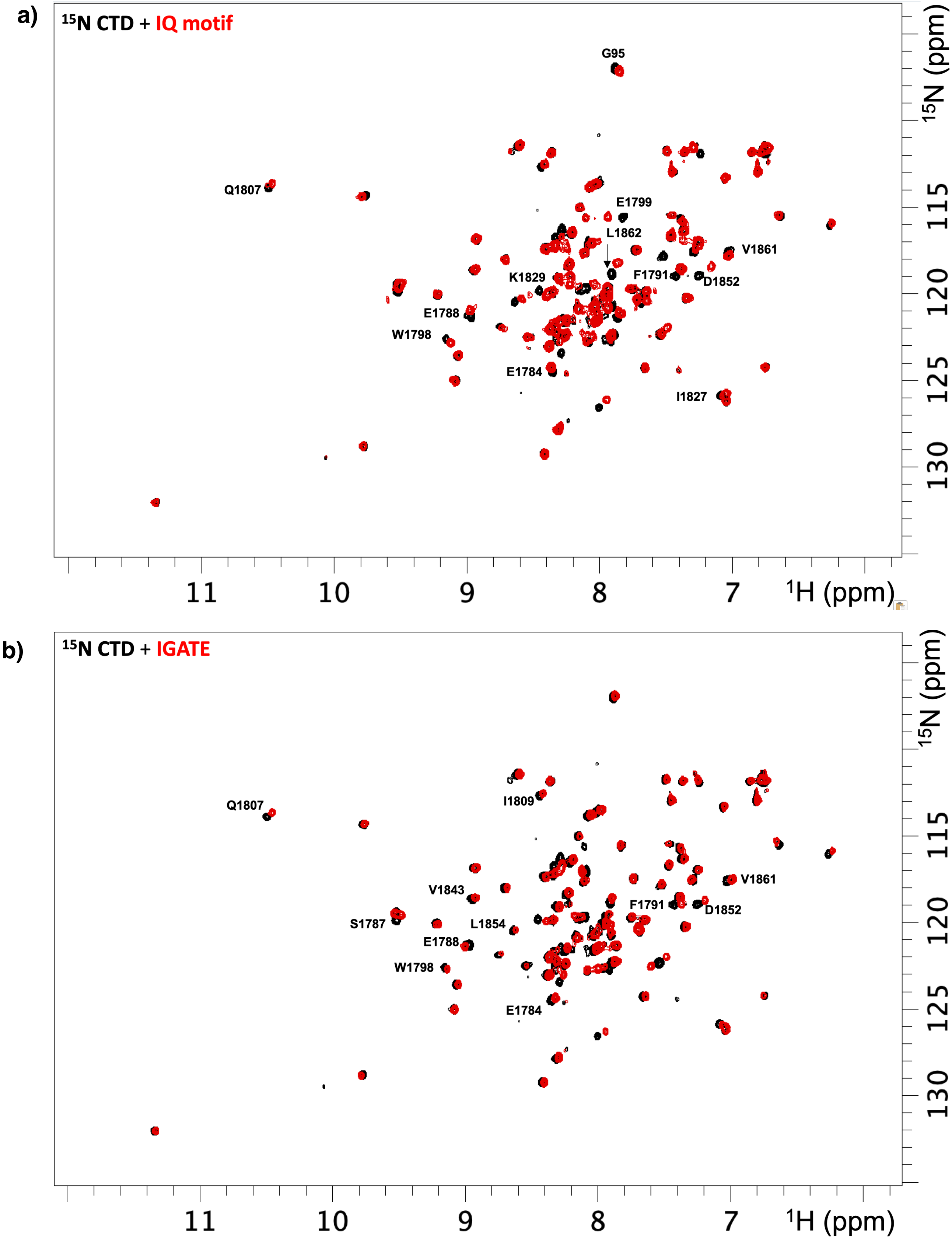

Figure S9. Overlays of ^{15}**N CTD** with **a)** IQ motif peptide **b)** IGATE peptide. The high degree of similarity between these spectra were consistent with a CTD engaging the IQ motif. and IGATE in a similar manner with the same binding interface.

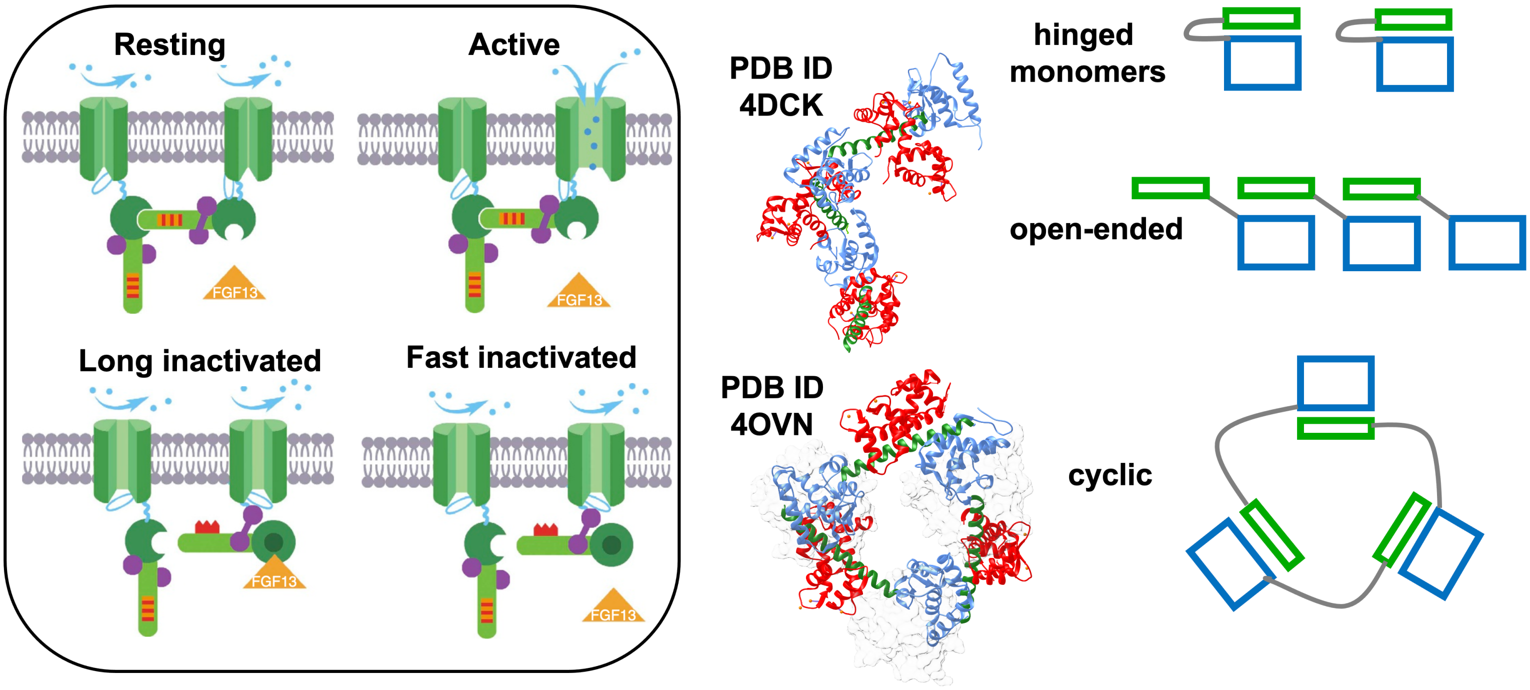

Figure S10. Previously posited mechanism for modification of Nav1.5 gating by Ca^{2+} CaM (Left) (Gabelli *et al* 2014) that is inconsistent with NMR data from this study. Crystal packing for CTD-IQ CaM structures in the absence (top) and presence (bottom) of FGF are consistent with domain swapped configurations (right). **CaM** (red), **CTD** (Blue), **IQ motif** (green).

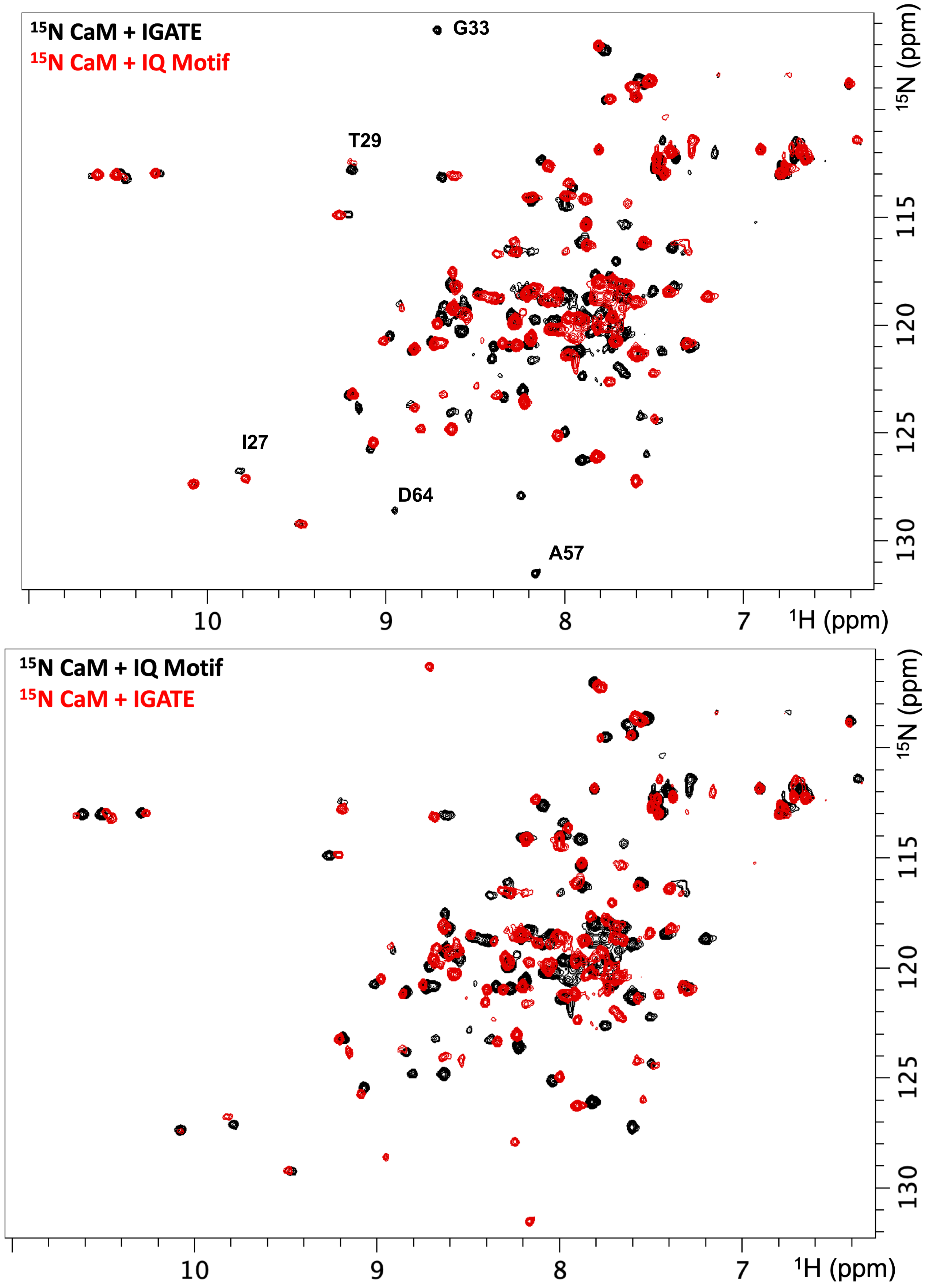

Figure S11. Overlays of HSQC ^{15}N CaM-IGATE on top of ^{15}N CaM-IQ motif (top) and ^{15}N CaM-IQ motif on top of ^{15}N CaM-IGATE (bottom). Differences between these spectra were consistent with the IGATE construct providing a more stable binding site interface for the CaM-N domain interaction in the presence of Ca^{2+}.

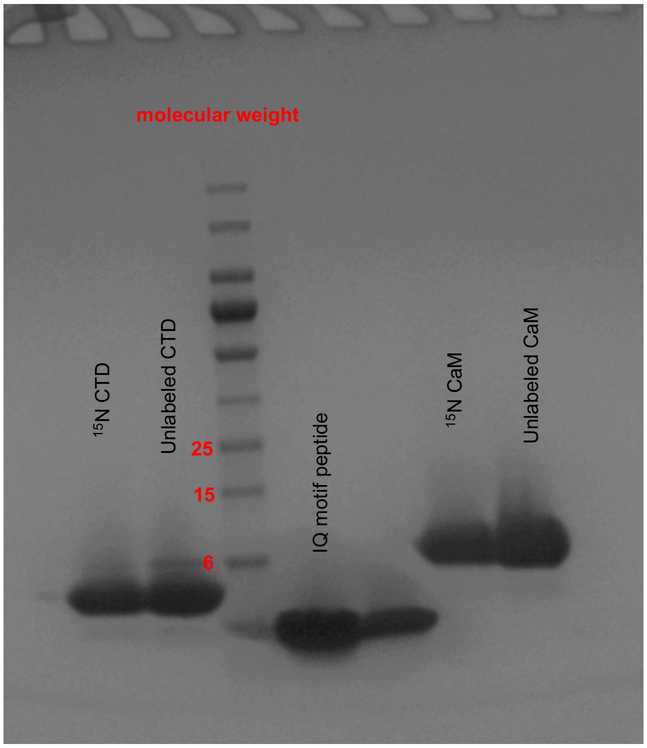

Figure S12. SDS gel of purified human protein used in NMR experiments.
